# How the magnitude and precision of pain predictions shape pain experiences

**DOI:** 10.1002/ejp.4769

**Published:** 2024-12-13

**Authors:** Suzanne M. J. C. Derksen, Maria Konttinen, Anastasiia Myronenko, Ben Seymour, Kaya J. Peerdeman

**Affiliations:** ^1^ Health, Medical and Neuropsychology Leiden University Leiden the Netherlands; ^2^ Nuffield Department of Clinical Neurosciences University of Oxford Oxford UK

## Abstract

**Background:**

In Bayesian models including predictive processing, the magnitude and precision of pain expectancies are key determinants of perception. However, relatively few studies have directly tested whether this holds for pain, and results so far have been inconclusive. Here, we investigated expectancy effects on pain experiences and associated affective responses.

**Methods:**

In two studies, healthy participants (*n* = 30 in each) received painful electrical stimuli preceded by explicit pain predictions. In study 1, the magnitude of pain predictions and administered pain intensities were varied. In study 2, the magnitude and precision of pain predictions were varied, while administered pain intensity was kept constant. Experienced pain intensity was the primary outcome in both studies.

**Results:**

Pain experiences assimilated towards both under‐ and overpredictions of pain. In study 1, however, effects were small, if present at all, for non‐painful stimuli and effects were not necessarily larger with predictions of greater magnitude. In study 2, assimilation of pain experiences appeared regardless of precision level, while no significant effects on EMG eyeblink startle responses were observed. Moreover, under‐ and overpredictions caused disappointment and relief, respectively, with greater disappointment upon precise than imprecise predictions.

**Conclusions:**

The influence of pain predictions on pain might be more complex than assumed in simple instantiations of current theoretical frameworks, with no systematically stronger assimilation of pain experiences to larger and more precise predictions. Since overpredictions are associated with relief, but underpredictions with disappointment, these findings underline the importance of providing correct predictions when preparing for upcoming painful procedures.

**Significance Statement:**

Our work supports, challenges, and extends the application of Bayesian and predictive processing frameworks to the influence of pain predictions on pain. Under‐ and overpredictions of pain yielded assimilation of pain experiences, but assimilation was not systematically stronger with larger prediction errors or greater precision. Moreover, under‐ and overpredictions resulted in disappointment and relief, respectively. This research signifies the importance of establishing accurate predictions of pain in clinical practice.

## 1 INTRODUCTION

Pain experiences are readily altered by expectancies (Büchel et al., 2014; Kirsch, 1985; Peerdeman et al., 2016). The importance of this phenomenon for clinical practice is underlined by a large body of research demonstrating placebo and nocebo effects on pain of which expectancies are the putative core mechanism (Evers et al., 2018, 2021). Generally, pain experiences have been found to be biased towards expectancies, known as assimilation (Blythe et al., 2022; Thomaidou et al., 2023). As such, upon underpredictions of pain (when anticipating less pain than actually encountered) experienced pain intensity may be reduced, while overpredictions of pain (when anticipating more pain than actually encountered) may increase pain intensity. The influential Bayesian models including predictive processing consider both the magnitude and precision of expectancies to be key determinants of their effects on perception (Büchel et al., 2014; Ongaro & Kaptchuk, 2019; Tabor & Burr, 2019). These frameworks pose that larger expectancies (i.e. larger difference between expectation and sensory input; larger prediction error) and more precise expectancies (i.e. more certain) yield stronger assimilation effects on pain. Some experimental studies confirm this (Grahl et al., 2018; Hoskin et al., 2019; Pollo et al., 2001). However, others show it might not always hold true. Strong underpredictions have been found to not result in more pain relief than moderate underpredictions (Hird et al., 2019; Peerdeman et al., 2021), and a recent meta‐analysis did not indicate any consistent influence of uncertainty on pain (Pavy, Zaman, Van Den Noortgate, et al., 2024).

Additionally, in their simplest form and as usually applied, Bayesian models including predictive coding have been less concerned with the emotions associated with unmet predictions of pain. However, they may result in disappointment, undermine trust, or induce pain‐related fear and increase anxiety, especially when substantially more pain is experienced than was expected (Arntz et al., 1991; Arntz & Hopmans, 1998; Campbell & Guy, 2007; Herruer et al., 2015; Husain & Lee, 2015; Peerdeman et al., 2021). The applicability of expectancy effects in clinical practice may be further challenged by large interindividual differences (e.g. in anxiety, optimism, interoceptive awareness, variability in pain reporting) that may influence pain experiences (Horing et al., 2014; Treister et al., 2019). Further research into how pain predictions shape pain experiences is required to test and expand dominant theories and inform on how healthcare providers can effectively harness expectancy effects to optimize patient outcomes.

This paper outlines 2 experimental studies that tested core hypotheses and boundaries of Bayesian, predictive coding models. In cued pain tasks, we manipulated pain predictions prior to delivering electrical stimuli and assessing experienced pain intensity. Primarily, we aimed to examine whether pain experiences assimilate towards pain predictions, and specifically how this is affected by the magnitude of the prediction (or size of the prediction error) (study 1) and the precision of the prediction (study 2). Secondarily, as pain intensity is only one aspect of patients' experiences, this project goes beyond these frameworks by also examining affective responses. Finally, effects on the startle reflex (study 2) and moderation by individual characteristics are explored.

## 2 METHODS

### 2.1 Methods study 1

In Study 1, the magnitude of pain predictions and electrical pain stimulus intensities were systematically varied to create under‐ and overpredictions (prediction errors) of different sizes, as well as correct predictions. Our primary hypothesis was that pain experiences would assimilate towards pain predictions, in line with Bayesian models (Büchel et al., 2014; Ongaro & Kaptchuk, 2019; Tabor & Burr, 2019), but that predictions offering the largest under‐ or overprediction of upcoming pain would not yield the largest assimilation effects (Hird et al., 2019; Peerdeman et al., 2021). Secondarily, we hypothesized that expectancies offering the largest under‐ or overprediction of upcoming pain would result in the strongest relief or disappointment, respectively. Lastly, we explored the possible moderation of the prediction effects on pain by individual characteristics (i.e. variability of pain reporting, anxiety, optimism, interoceptive awareness, and pain vigilance).

The study was approved by Leiden University's Psychology Research Ethics Committee (2021‐11‐15‐K.J.Peerdeman‐V1‐3547) and preregistered at the Open Science Framework (DOI: 10.17605/OSF.IO/W3PUZ, https://osf.io/w3puz/).

#### 2.1.1 Participants

The sample size was determined based on the sample sizes used in previous studies using a comparable design and analytical approach (Fazeli & Büchel, 2018; Geuter et al., 2017; Hird et al., 2019; Hoskin et al., 2019; Yoshida et al., 2013; Zaman et al., 2017) as a power analysis for our multi‐factorial within‐subjects study design is complex. These studies indicate that sufficient power to find meaningful, moderate‐sized, effects should be obtained with data of 30 participants per study. Healthy participants were recruited via flyers in faculty buildings in Leiden, The Hague, and Delft, and through social media channels. They signed up through the university's standard participant management tool (SONA) and were sent the participant information letter via e‐mail. Participants had to be between 18 and 35 years, fluent in English (written and spoken), and able to give informed consent. To ensure participants' safety and data quality, participants were excluded if they suffered from a severe physical or psychiatric condition that could possibly interfere with the study protocol (e.g. cardiac disease, DSM‐V diagnosis, severe hearing or vision problems), had a history of chronic (≥3 months) pain, experienced current pain (>1 on a 0–10 scale), had injuries to the non‐dominant arm on the day of testing, were fitted with an implanted electronic device, currently used medication (e.g. analgesic within 24 h prior to testing), used alcohol (>1 glass) or drugs within 24 h prior to testing, were pregnant or breastfeeding, or in case of unsuccessful pain calibration (i.e. not experiencing high pain at or below the maximum stimulus intensity).

#### 2.1.2 Electrical pain evocation, assessment and calibration

Pain was evoked with electrical stimulations delivered for a duration of 1000 ms via electrodes by a constant current stimulator (Digitimer DS5 2000, Digitimer Ltd., Welwyn Garden City, UK). The DS5 was set to an input voltage of 5 V and a maximum output current of 25 mA, although no stimuli above 10 mA were provided. A custom‐built Signal Generator was used to connect the DS5 to the PC. Spectra 360 electrode gel was applied to both disks of a bar stimulating electrode (two stainless steel round 10 mm electrode‐surfaces at a spacing of 30 mm, Digitimer BARR0026). Transpore White tape was used to attach the bar electrode to the volar forearm approximately 3 cm from the elbow crease.

Experienced pain intensity during the electrical stimuli was rated on a digital, horizontally‐presented 11‐point numerical rating scale (NRS) ranging from 0 (‘no pain at all’) to 10 (‘most intense pain imaginable’). Participants could tick one of the boxes below each number (integers only) and were informed about the meaning of additional scale points (2 = ‘low pain’, 4 = ‘moderate pain’, 6 = ‘high pain’, 8 = ‘very high pain’).

The electrical pain calibration procedure consisted of three steps. Step 1 was the familiarization phase. Participants received an ascending series of electrical stimuli, starting at 0.5 mA and increasing with steps of 0.5 mA. Participants indicated verbally the first time they felt the stimulation (perception threshold), when the stimulation first became painful (pain perception threshold), and the maximum pain intensity they could tolerate (pain tolerance level). Stimulation was stopped at the pain tolerance level or when the maximum of 10 mA was reached. In Step 2, participants received the same ascending series of stimuli as in Step 1. After each stimulus, participants now rated experienced pain intensity on the NRS. The stimulation was stopped as soon as participants gave a rating of 8 (i.e. very highly painful) on the NRS or when the maximum of 10 mA was reached. Participants who did not rate 10 mA with at least 6 were excluded. The experimenter informed participants that the last stimulus would be the highest to be used throughout the experiment alongside lower intensity stimuli. In step 3, a random series of stimuli was presented. The OpenSesame software randomly selected 30 intensities between 0.5 mA and the highest intensity that participants experienced in step 2. After each stimulus, participants again rated experienced pain intensity on the NRS. Based on these ratings, the software selected intensities corresponding to ‘no pain at all’ (0 on the NRS), ‘moderate pain’ (4 on the NRS), and ‘very high pain’ (8 on the NRS) by fitting a Weibull function to the participants' ratings for all different stimulus intensities given in step 3 (Weibull, 1951; Yoshida et al., 2013; Zaman et al., 2017). To ensure a good fit, differences of at least 0.5 mA between each intensity, and the selection of non‐painful yet perceptible intensities for the non‐painful stimulus, the fitted function was plotted on the provided stimulus intensities and ratings. This was inspected by the experimenter and manual adjustments were made when needed.

#### 2.1.3 Uncued pain task

An uncued pain task was used to assess variability of pain intensity ratings. By presenting pain stimuli without cues, we aimed to investigate how participants perceived and rated pain intensity under unpredictable conditions. This task consisted of 2 separate blocks. In each block, the three stimulus intensities selected during calibration (evoking no, moderate, and very high pain) were each presented three times, in pseudorandom order (i.e. 9 trials per block). For each individual participant, the R2 of a linear model regression with stimulus intensity (i.e. no, moderate, and very high pain, as indicated by ratings of 0, 4, and 8 on the NRS during calibration, respectively) as predictor and pain rating as dependent variable was calculated across all trials of the first block of the uncued pain task to indicate variability. Lower R2 scores indicated higher variability. This approach was based on the work of Treister and colleagues (Treister et al., 2017).

#### 2.1.4 Cued pain task—magnitude of predictions

We designed a cued pain task to assess the influence of pain predictions on pain experiences. Participants received electrical pain stimuli of three different individually calibrated intensities: no pain (1.37 ± 0.87 mA), moderate pain (4.08 ± 1.67 mA), or very high pain (5.67 ± 2.02 mA). Each stimulus was preceded by a lexical cue indicating a precise prediction of the pain intensity to be expected: ‘No pain’, ‘Low pain’, ‘Moderate pain’, ‘High pain’, and ‘Very high pain’. Participants were informed that some cues were a correct prediction of the upcoming intensity, whereas others were an under‐ or overprediction, hereby, no deception was used.

The task consisted of 2 counterbalanced blocks of 34 trials each (trials were presented in a pseudorandom order, 68 trials in total). Each block was preceded by 2 practice trials with correctly predicted non‐painful stimuli to familiarize participants with the procedure. In each block, about half of the stimuli were correctly predicted and half were incorrectly predicted, with an equal amount of underpredicted and overpredicted stimuli, to ensure participants considered the cues as relevant predictors of stimulus intensity. Also, different magnitudes of prediction errors were presented equally often, despite the extremes naturally occurring less frequently, to ensure sufficient data for comparisons. See Table 1 for an overview of the frequency with which each unique cue and stimulus combination is presented.

**TABLE 1:** Overview of the frequency of each unique cue and stimulus combination in the cued pain task in study 1.

Cue/pain prediction	Stimulus intensity	Prediction error	Number of trials per block
No pain	8	‐8	2
Low pain	8	‐6	2
Moderate pain	8	−4	1
No pain	4	−4	1
Low pain	4	−2	1
High pain	8	−2	1
No pain	0	0	6
Moderate pain	4	0	6
Very high pain	8	0	6
Low pain	0	2	1
High pain	4	2	1
Moderate pain	0	4	1
Very high pain	4	4	1
High pain	0	6	2
Very high pain	0	8	2

*Note*: Each cue corresponds to a numerical value: ‘No pain’ = 0, ‘Low pain’ = 2, ‘Moderate pain’ = 4, ‘High pain’ = 6, and ‘Very high pain’ = 8. Each stimulus intensity corresponds to the rated pain intensity during the calibration procedure. The prediction error is the numerical difference between the cue and stimulus intensity and represents the numerical underprediction, overprediction, or correct prediction. The number of trials is the number of times each unique cue and stimulus combination was presented during each block of the cued pain task.

**FIGURE 1:**
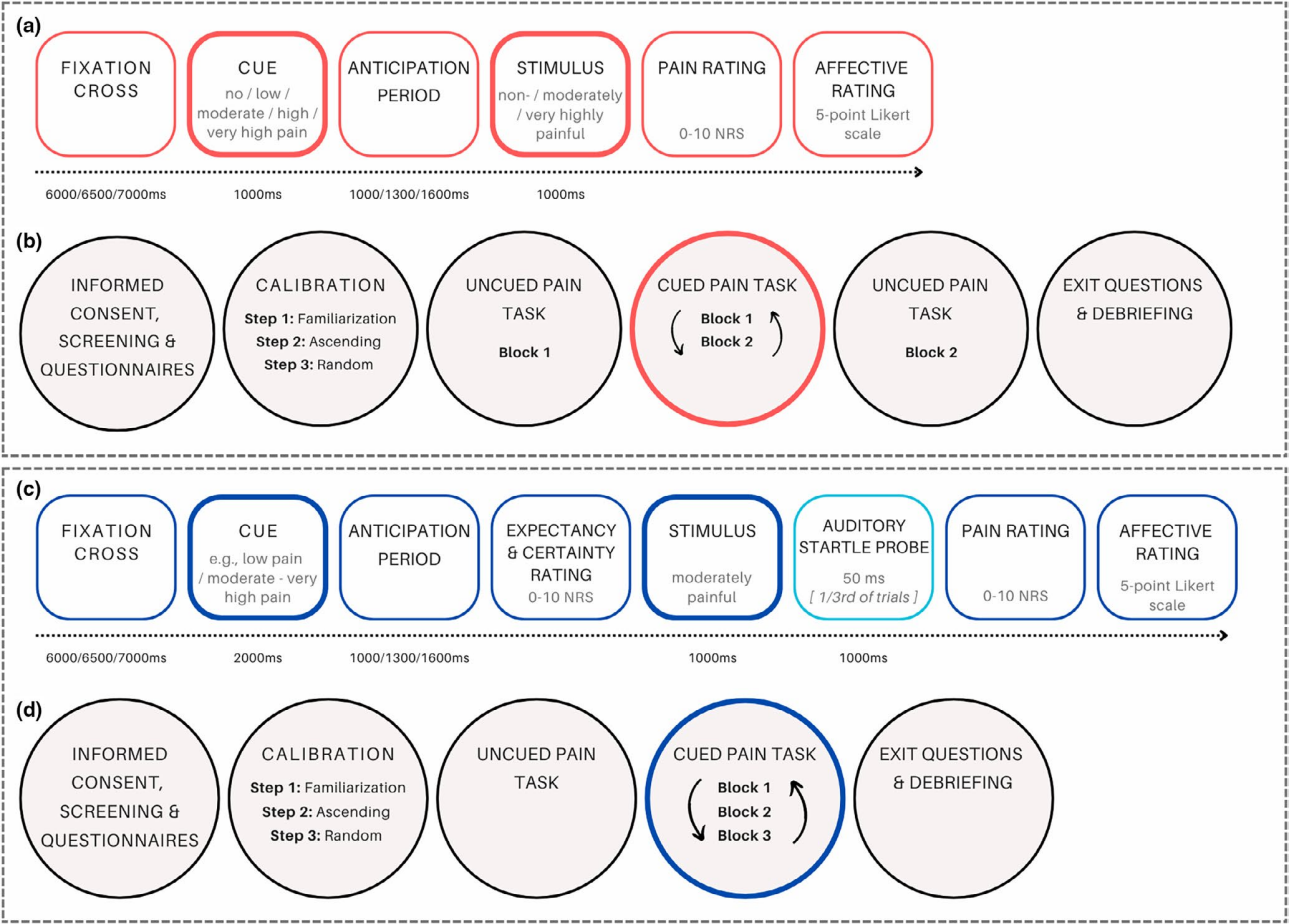
Overview of the trial presentation of the cued pain task and the overall study procedures per study. (a) Trial presentation study 1, showing the trial elements (e.g. cue, stimulus) in chronological order, with a brief specification (i.e. exact cues given, calibrated stimulus intensity, rating scales) and the duration indicated below in milliseconds, unless timing was determined by participants' response. (b) Overall study procedure study 1, presenting the study elements in chronological order from informed consent to debriefing. (c) Trial presentation study 2, showing the trial elements in chronological order, with a brief specification and the duration indicated below in milliseconds, unless timing was determined by participants' response. Differences with study 1 were in the exact cues presented, calibrated stimulus intensity, and the assessment of auditory startle responses after the stimulus in 1/3rd of the trials. (d) Overall study procedure study 2, presenting the study elements in chronological order from informed consent to debriefing. Different than in study 1, the uncued pain tasks consisted of a single block, and the cued pain task consisted of 3 counterbalanced blocks. Note, the circular arrows indicate counterbalancing of blocks in the cued pain tasks. NRS, numerical rating scale.

The trial procedure is depicted in Figure 1a. Each trial started with a fixation screen for either 6000 ms, 6500 ms, or 7000 ms to control for predictability of timing in the experimental task. Then, the cue was presented in purple font on the screen for 1000 ms. An anticipation period followed for 1000 ms, 1300 ms, or 1600 ms. A variable anticipation period, rather than fixed, was chosen to reduce a sense of routine and consequently a decrease in attention and motivation, which could potentially affect the quality of responses. After the anticipation period, the stimulus was administered (1000 ms), upon which participants rated their pain intensity on the NRS, which was confirmed by clicking ‘Enter’. To prevent missing data, no time limit was set for providing a response. Finally, participants rated their affective responses to experiencing the predicted pain stimuli on a digital 5‐point Likert scale (0 = very disappointed, 1 = disappointed, 2 = neutral, 3 = relieved, 4 = very relieved), which was also confirmed by clicking ‘Enter’.

#### 2.1.5 Questionnaires

Dispositional optimism was assessed with the Revised Life Orientation Test (LOT‐R) (Scheier et al., 1994). A high total score indicates high optimism. To assess state and trait anxiety, we used the State–Trait Anxiety Inventory (STAI)—short‐form state scale (STAI‐S) (Marteau & Bekker, 1992) and full‐form trait scale (STAI‐T), respectively, with high total scores indicating high anxiety (Spielberger et al., 1999). Attention to pain and changes in pain was assessed with the Pain Vigilance and Awareness Questionnaire (PVAQ) (Roelofs et al., 2003), with a high total score reflecting greater vigilance. Item 11 of the PVAQ was erroneously missing, consequently the total score ranged from 0 to 75 (instead of 0 to 80). Interoceptive awareness was measured with 4 subscales of the Multidimensional Assessment of Interoceptive Awareness (MAIA‐2) (Mehling et al., 2018), with a higher total score indicating more awareness of bodily sensation. The 4 subscales used were ‘Not‐Distracting’ and ‘Not‐Worrying’, where a higher score indicates increased emotional reactions and attentional response to sensations, ‘Self‐Regulation’, where a higher total score indicates increased awareness of mind–body integration, and ‘Trust’, where a higher total score reflects increased level of trusting bodily sensations. Finally, two exit questions assessed how focused participants were on their sensations during the stimuli and to what extent participants regarded the pain predictions as informative predictors of the intensity of the electrical stimuli (0 = not at all, 1 = slightly, 2 = moderately, 3 = very, and 4 = extremely).

#### 2.1.6 General procedure

Data were collected at a standard psychophysiology laboratory at the Faculty of Social and Behavioural Sciences, Leiden University, Leiden, the Netherlands from December 2021 to February 2022. See Figure 1b for an overview of the procedures. Upon entering the lab, participants were fully informed about the study details following a standard protocol. Potential questions were answered, after which both the participant and the experimenter signed the informed consent form. Next, eligibility was ensured through screening, and afterwards, participants filled in the questionnaires. Then pain calibration started, with the first block of the uncued pain task following directly. After a 3‐min break, the cued pain task started, with 5‐min breaks between the blocks of the task. Subsequently, the second block of the uncued pain task followed. Finally, participants filled in exit questions and read the debriefing. Participants were compensated according to the university's standard rates for the time invested in participating in each study. The full study procedures were 1 h and 45 min.

All cues, electrical stimuli, rating scales, probes, and breaks were triggered by OpenSesame (version 3.3.3) (Mathôt et al., 2012). Qualtrics (Provo, UT) was used for questionnaires and presenting the debriefing to participants. Experimenter notes were taken in a separate form in Qualtrics.

#### 2.1.7 Statistical analyses

All statistical analyses were run in Rstudio (version 2022.07.0; R version 4.2.1). The standard alpha = 0.05 criterion was used for determining if test results indicate statistically significant differences, unless otherwise specified. Although normality may be assumed with the current sample size (Field et al., 2012, section 5.5), the assumption of normality was checked using box plots, histograms, and Q‐Q plots for all analyses to detect severe deviations. The assumption of sphericity was checked using Mauchly's test and Greenhouse–Geisser correction was applied if violated. For analyses of variance, generalized eta squared (*η*_*g*_^2^) was computed, with 0.01, 0.06, and 0.14 signifying small, medium, and large effects, respectively (Lakens, 2013). For pairwise comparisons, Cohen's *d* was computed, with 0.2–0.3, 0.5, and ≥0.8 signifying small, medium, and large effects, respectively (Cohen, 1988).

For the primary hypothesis, a 5 (pain prediction: no pain, low pain, moderate pain, high pain, very high pain) × 3 (stimulus intensity: no pain, moderate pain, very high pain) within‐subjects analysis of variance (ANOVA) was used with pain intensity ratings as the dependent variable. Pain intensity ratings were calculated as an average across trials of each of the 15 unique cue and stimulus combinations. A significant interaction effect was followed up by pairwise comparisons of the over−/underpredictions with the correct prediction for each stimulus intensity level separately (when preparing the analyses this was deemed more informative on the effects of the over‐ and underprediction of pain than the preregistered approach of comparing increasingly large predictions). Controlling for multiple testing was done with a Bonferroni correction, by dividing the alpha with the number of analyses (*n* = 4) per stimulus intensity level. Since the assumption of normality was clearly violated for these primary analyses, with many participants reporting no pain (0 on the NRS) for the non‐painful stimulus, and also considering the bounded nature of the data (ranging between 0 and 10), we ran additional zero‐inflated beta regression analyses. The R‐package gamlss was used to test a null‐model including the intercept, a model also including the main effect of stimulus intensity, a model also including the main effects of stimulus intensity and pain prediction, and a model also including the main effects and interaction of pain prediction and stimulus intensity. Likelihood ratio tests were used to compare the consecutive tests. Zero‐inflated beta regression analyses were also run for the pairwise comparisons of the over−/underpredictions with the correct prediction for the non‐painful stimulus, while regular beta regression analyses were used for these pairwise comparisons for the moderately and very highly painful stimuli. For these comparisons, a model containing the pairwise comparison was compared to a null‐model including the intercept only using likelihood ratio tests, for each stimulus intensity separately.

For the secondary hypothesis, the same 5 × 3 repeated measures ANOVA and subsequent approach for pairwise comparisons were used, but with the affective response as the dependent variable.

To test the exploratory hypotheses regarding the questionnaire scores and pain rating variability moderating the prediction effects on pain experiences, each variable was separately added as a covariate to the primary ANOVA and the interactions between each potential moderator and the pain predictions and/or stimulus intensity were examined. We had also preregistered exploratory analyses of the effects of the predictions on the confidence in pain ratings (rated on an NRS from not certain at all to very certain about the pain rating) and reaction times for providing pain ratings. However, due to methodological issues these data proved to be of insufficient quality to be analysed or reported.

### 2.2 Methods study 2

In study 2, both the magnitude and precision of pain predictions were systematically varied, while pain stimulus intensity was kept stable. Our primary hypothesis was that pain experiences would assimilate towards pain predictions, but that the assimilation effects to under‐ and overpredictions would depend on the precision of the pain prediction, where imprecise predictions would yield smaller effects compared to precise predictions (Büchel et al., 2014; Grahl et al., 2018; Hoskin et al., 2019; Ongaro & Kaptchuk, 2019; Pollo et al., 2001; Tabor & Burr, 2019). Secondarily, we hypothesized that precise rather than imprecise pain predictions would result in the strongest relief or disappointment upon over‐ or underprediction, respectively. We also explored if eyeblink startle responses, as indicator of pain‐related fear, would be greater upon experienced over‐ or underpredictions than correct predictions of pain, particularly when precise. The startle reflex was chosen due to this measurement being non‐conscious. We additionally checked if the predictions manipulated expectations as intended, by examining pain expectations and certainty thereof. Lastly, we explored the possible moderation of the prediction effects on pain by individual characteristics (i.e. variability of pain reporting, anxiety, optimism, intolerance of uncertainty, and pain vigilance).

The study was approved by Leiden University's Psychology Research Ethics Committee (2022‐03‐15‐K.J. Peerdeman‐V2‐3829) and preregistered at the Open Science Framework (DOI: 10.17605/OSF.IO/FX96A, https://osf.io/fx96a/).

#### 2.2.1 Participants

The sample size determination and eligibility criteria in study 2 identical to those in study 1.

#### 2.2.2 Electrical pain calibration

Steps 1 and 2 of the pain calibration procedure in study 2 were almost identical to study 1, with one difference. In study 1 we found that in step 2 the ratings of the highest stimuli were on average 7.1 on the NRS instead of the intended 8 during the subsequent step and the cued pain task. Therefore, participants received one more stimulus after a rating of 8 (if possible) in step 2 of study 2. For the same reason, participants who did not rate 10 mA with at least 7 were excluded, and the procedures were stopped. The experimenter told participants that the last stimulus would be the highest to be used throughout the experiment alongside lower intensity stimuli. In step 3, the same approach was used as in study 1, but now intensities corresponding to ‘low pain’ (2 on the NRS) and ‘high pain’ (6 on the NRS) were additionally selected.

#### 2.2.3 Uncued pain task

An uncued pain task was used to assess variability of pain intensity ratings. Different than in study 1, this task consisted of 1 block, in which participants received 30 electrical stimuli in a pseudorandom order. The five stimulus intensities selected during calibration were each presented six times.

#### 2.2.4 Cued pain task—magnitude and precision of predictions

We designed a cued pain task similar to the cued pain task of study 1, but now to assess the influence of both the magnitude and precision of pain predictions on pain experiences. As in study 1, pain predictions preceded electrical pain stimuli. Differences in the precise cues, stimulus intensities, block and trial procedures are highlighted below.

In study 2, both precise cues (‘Low pain’, ‘Moderate pain’, and ‘High pain’) and imprecise cues (‘No pain to moderate pain’, ‘Low pain to high pain’, and ‘Moderate pain to very high pain’) were presented. Only moderately painful stimuli (4 on NRS) were administered to compare precise and imprecise over‐ and underpredictions with similar (average) prediction errors. The intensity of the electrical stimuli varied with minus 0.1 mA or plus 0.1 mA from the individually calibrated intensity (4.32 ± 1.65 mA) to reduce the chance that participants would become aware all stimuli were of moderate intensity.

The task consisted of 3 counterbalanced blocks of 18 trials each (trials were presented in pseudorandom order, 54 trials in total). See Table 2 for an overview of the frequency of each unique cue and stimulus combination. The first block was preceded by 2 practice trials consisting of moderately painful stimuli that were correctly predicted. The second and third blocks were preceded by only 1 practice trial.

**TABLE 2:** Overview of the frequency of each unique cue and stimulus combination in the cued pain task in study 2.

Cue/pain prediction	Stimulus intensity	Prediction error	Precision	Number of trials per block
No pain to moderate pain	4	−2	Imprecise	3
Low pain	4	−2	Precise	3
Low pain to high pain	4	0	Imprecise	3
Moderate pain	4	0	Precise	3
Moderate pain to very high pain	4	2	Imprecise	3
High pain	4	2	Precise	3

*Note*: Each precise cue corresponds to an exact numerical value where ‘Low pain’ = 2, ‘Moderate pain’ = 4, and ‘High pain’ = 6. Each imprecise cue corresponds to an approximate numerical value where ‘No pain to moderate pain’ ≈ 2, ‘Low pain to high pain’ ≈ 4, and ‘Moderate pain to very high pain’ ≈ 6. The stimulus intensity corresponds to the rated pain intensity during the calibration procedure (±0.1 mA). The prediction error is the (approximate) numerical difference between the cue and stimulus intensity and represents the average numerical underprediction, overprediction, or correct prediction, with imprecise cues always including a correct prediction. The number of trials is the number of times each unique cue and stimulus combination was presented during each block of the cued pain task.

The trial procedure is depicted in Figure 1c. Each trial started with a fixation screen for either 6000 ms, 6500 ms, or 7000 ms. Then the cue was presented (2000 ms) and an anticipation period of either 1000 ms, 1300 ms, 1600 ms followed. Next, participants rated how much pain they expected on the same NRS as pain intensity and how certain they were of the expectation on an 11‐point NRS where 0 means not certain at all and 10 means very certain. Upon confirming their response, the electrical pain stimulus followed (1000 ms). In 18 trials (one‐third of all trials, in pseudorandom order) an auditory startle probe was presented. The probes were presented during a 1000 ms time frame, 500 ms after the stimulus. Finally, participants rated their pain intensity and affective response.

#### 2.2.5 Electromyography (EMG) eyeblink responses

EMG eyeblink responses to auditory startle probes were measured in one‐third of the trials. The auditory startle probe was a burst of white noise for a duration of 50 ms with an instantaneous rise time to an approximately 98 dB calibrated peak (Blumenthal et al., 2005), presented via headphones (Sennheiser HD206). To measure orbicularis oculi EMG activity, we used two EL254S Ag‐AgCl BIOPAC electrodes (4 mm recording diameter) on the orbicularis oculi below the right eye and one EL654 Ag‐AgCl BIOPAC ground electrode (4 mm recording diameter) on the forehead, following the Blumenthal et al. guidelines (Blumenthal et al., 2005). All electrodes were filled with Signa electrode gel. Before attaching the electrodes, the experimenter gently exfoliated the skin with NuPrep scrub. The signal was recorded with a BIOPAC Electromyogram Amplifier EMG100C and MP150 module and AcqKnowledge software (version 5.0; BIOPAC Systems Inc., Goleta, CA) at a sampling rate of 2000 Hz, with a low‐pass filter of 500 Hz and a high‐pass filter of 10 Hz.

#### 2.2.6 Questionnaires

In study 2, as in study 1, dispositional optimism, state and trait anxiety and attention to pain and changes in pain were assessed with the same questionnaires, as was informativeness of the predictions. In study 2, unlike study 1, intolerance of uncertainty was measured with the 12‐item Intolerance of Uncertainty Scale (IUS‐12) (Carleton & Asmundson, 2007). A higher total score on the IUS‐12 indicates higher levels of intolerance of uncertainty.

#### 2.2.7 General procedure

Data were collected from April to May 2022. In study 2, the general procedure was largely the same as in study 1. See Figure 1d for an overview of the procedures. Different was that pain calibration was now followed by a 5‐min break, after which EMG electrodes were attached. Then the uncued and cued pain tasks specific for study 2 followed, with 3‐min breaks between the blocks of the latter task. The full study procedures took 2 h.

#### 2.2.8 Statistical analyses

The same general analysis approach was used in study 2 as in study 1. For study 2 specifically, manipulation checks were done for pain expectations and certainty thereof with two 3 (magnitude: under−/over−/correct prediction) × 2 (precision: precise versus imprecise) within‐subjects ANOVAs. A significant interaction effect was followed up by pairwise comparisons of the over−/underprediction with the correct prediction for each level of precision separately (Bonferroni correction was applied by dividing the alpha by the number of analyses per precision level, i.e. 2), as well as with pairwise comparisons of the precise versus imprecise cues for each level of magnitude (i.e. under−/over−/correct prediction), separately (this is a slight deviation from the preregistered pairwise comparisons, to get more comprehensive insights). For the primary and secondary hypotheses, we used the same approach with pain intensity ratings or affective responses as dependent variable. Ratings were calculated as an average across trials of each of the 6 unique cues. To test the exploratory research questions regarding questionnaire scores and pain rating variability moderating the prediction effects on pain experiences, each variable was separately added as a covariate to the primary ANOVA and the interactions between each potential moderator and the magnitude and/or precision of the pain predictions were examined.

All raw EMG data were visually inspected, and any technical artefacts or other abnormalities were identified and removed if needed. Eyeblink auditory startle responses were preprocessed in the PhysioData Toolbox for Matlab (Version 0.6.3) (Sjak‐Shie, 2022). The raw EMG signal was digitized at 1000 Hz. First, the raw EMG data were prefiltered with a high‐pass filter of 28 Hz, a low‐pass filter at 500 Hz, and a notch filter at 50 Hz, in line with van Boxtel's (2010) recommendations for enhancing signal clarity and minimizing artefacts. Next, the signal was rectified to convert all its values to positive magnitudes to prepare for smoothing. Smoothing was then applied, using a moving average approach to enhance the signal‐to‐noise ratio. This involved averaging adjacent data points within a 100 ms window with a Boxcar filter of 100 ms/Hz. Each startle trial was subsequently segmented into predefined epochs, in line with Aslaksen et al. (2016). Each trial started at probe onset and concluded 200 ms post‐probe onset, capturing the primary response period. A baseline epoch for each trial, serving as a reference for normalizing startle response magnitudes (Aslaksen et al., 2016), was delineated to start 100 ms before probe onset and end at probe onset. Startle responses were calculated by subtracting the average baseline level (mV) from peak amplitudes (mV) within the primary response period. Trials were excluded (labelled as reject trials) if the baseline level was higher than the peak amplitude within the primary response period response peak, the baseline measurement was noisy, or if the eyeblink response occurred outside the window of interest. The startle probe trials of each unique cue and stimulus combination were averaged for further analyses. To test the exploratory hypotheses regarding the prediction effects on EMG eyeblink startle responses, a 3 × 2 within‐subjects ANOVA was run as for the other dependent variables.

## 3 RESULTS

For both studies, descriptives (mean, SD) for each reported measure and detailed statistics of all analyses are presented in the supplementary material (Table S1–6 for study 1; Table S7–17 for study 2). Additionally, study materials, data, analysis scripts, and results files are openly available through the online archiving system DataverseNL at https://doi.org/10.34894/L0YWET.

### 3.1 Results study 1

#### 3.1.1 Participants

One participant was excluded during screening and two participants were excluded due to unsuccessful pain calibration. All other 30 participants completed their participation in the study (86.7% female; 13.3% male; M age = 20.5, SD = 2.6). Participants varied in the extent to which they considered the cues to be informative predictors of stimulus intensity (*n* = 3 not at all, 12 slightly, 8 moderately, 7 very), while most were at least very focused on their sensations during the stimuli (*n* = 22 very, 6 extremely) (Table S1).

#### 3.1.2 Effect of magnitude of pain prediction on pain intensity ratings

**FIGURE 2:**
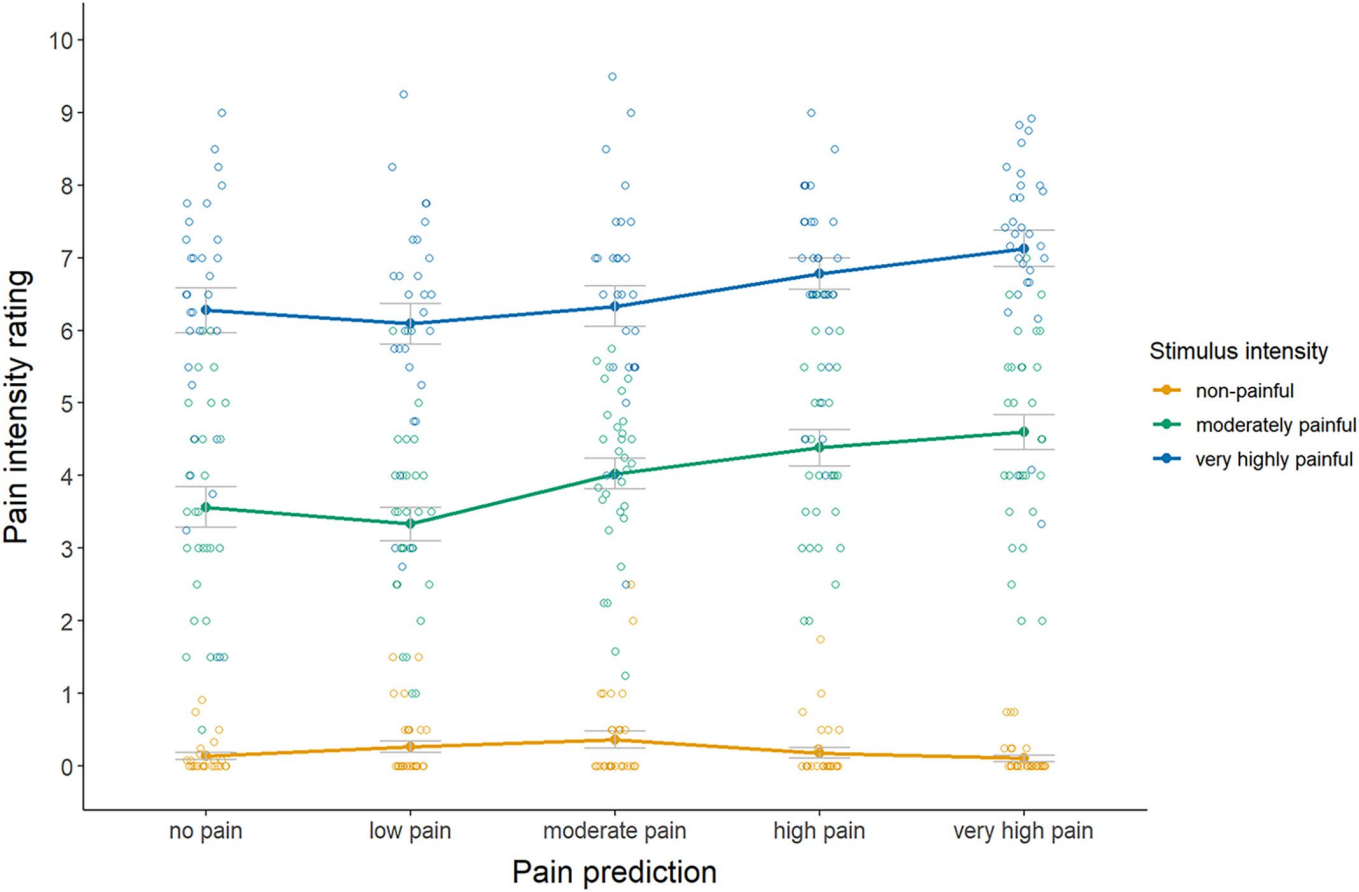
Mean pain intensity ratings (± standard error) for each cue and stimulus intensity (study 1). Pain intensity was rated on an 11‐point numerical rating scale, where 0 means no pain at all, 2 means low pain, 4 means moderate pain, 6 means high pain, 8 means very high pain, and 10 means the most intense pain imaginable.

For the primary hypothesis (see Figure 2; Table S2 for descriptives), results indicated a significant interaction effect between pain prediction and stimulus intensity on pain intensity ratings, *F*(5.16, 149.71) = 8.71, *p* ≤ 0.001, *η*_*g*_^2^ = 0.04. Follow‐up pairwise comparisons (see Table S4 for full test results, including estimated mean differences with 95% confidence intervals) for the non‐painful stimulus did not indicate significant assimilation of pain ratings upon any degree of overprediction as compared to correct prediction (prediction error (PE) = 2, *d* = 0.35; PE = 4, *d* = 0.41; PE = 6, *d* = 0.15; PE = 8, *d* = 0.17). For the moderately painful stimulus, overprediction yielded significant assimilation of pain ratings when this was moderate (PE = 4, *d* = 0.58), but not when this was small (PE = 2, *d* = 0.42), while both small and moderate underprediction yielded significant assimilation of pain ratings (PE = −2, *d* = 0.79; PE = −4, *d* = 0.51), as compared to correct prediction. For the very highly painful stimulus, moderate, large, and very large underprediction caused significant assimilation of pain ratings (PE = −4, *d* = 0.93; PE = −6, *d* = 1.24; PE = −8, *d* = 0.82), while small underprediction did not (PE = −2, *d* = 0.48), as compared to correct prediction. These findings indicate that for moderately and very highly painful stimuli, pain experiences tended to assimilate towards predictions, but not always significantly in cases of small prediction errors. Predictions did not significantly affect experiences of the non‐painful stimulus. Notably, assimilation was not necessarily larger with larger prediction errors.

Additional zero‐inflated beta regression analyses were run because of the many participants reporting no pain (0 on the NRS) for the non‐painful stimulus and the bounded nature of the data. These analyses also indicated a significant interaction effect between pain prediction and stimulus intensity on pain intensity ratings (likelihood ratio of the complete model vs. the model including only the main effects: *χ* ^2^ (8.10) = 28.03, *p* ≤ 0.001). In addition to the pairwise comparisons that were found to be significant with the original ANOVA, four additional pairwise comparisons were now observed to be significant. Specifically, for the non‐painful stimulus the comparisons now also indicated significant assimilation of pain ratings upon small and moderate overprediction as compared to correct prediction (PE = 2, *p* = 0.002, PE = 4, *p* ≤ 0.001). For the moderately painful stimulus, the small overprediction now also yielded significant assimilation of pain ratings (PE = 2, *p* = 0.002). For the very highly painful stimulus, small underprediction now also was found to cause significant assimilation of pain ratings (PE = −2, *p* = 0.004). These findings align with the size of the effects observed for the original analyses, with only truly small effects not being found to be statistically significant. These findings thus indicate that assimilation can occur for all stimuli, including in cases of small prediction errors, though these effects appear small.

Post‐hoc analyses examining possible time effects by examining the pain ratings averaged per block of the cued pain task indicated similar results during the first block that participants received as for the ratings averaged across blocks, but effects tended to be smaller for the second block (interaction effect first block *F*(4.72, 136.97) = 6.00, *p* ≤ 0.001, *η*_*g*_^2^ = 0.05; second block *F*(4.13, 119.67) = 3.32, *p* = 0.012, *η*_*g*_^2^ = 0.02; see Table S2 for descriptives and Table S5 for full test results pairwise comparisons).

#### 3.1.3 Effect of magnitude of pain prediction on affective response

**FIGURE 3:**
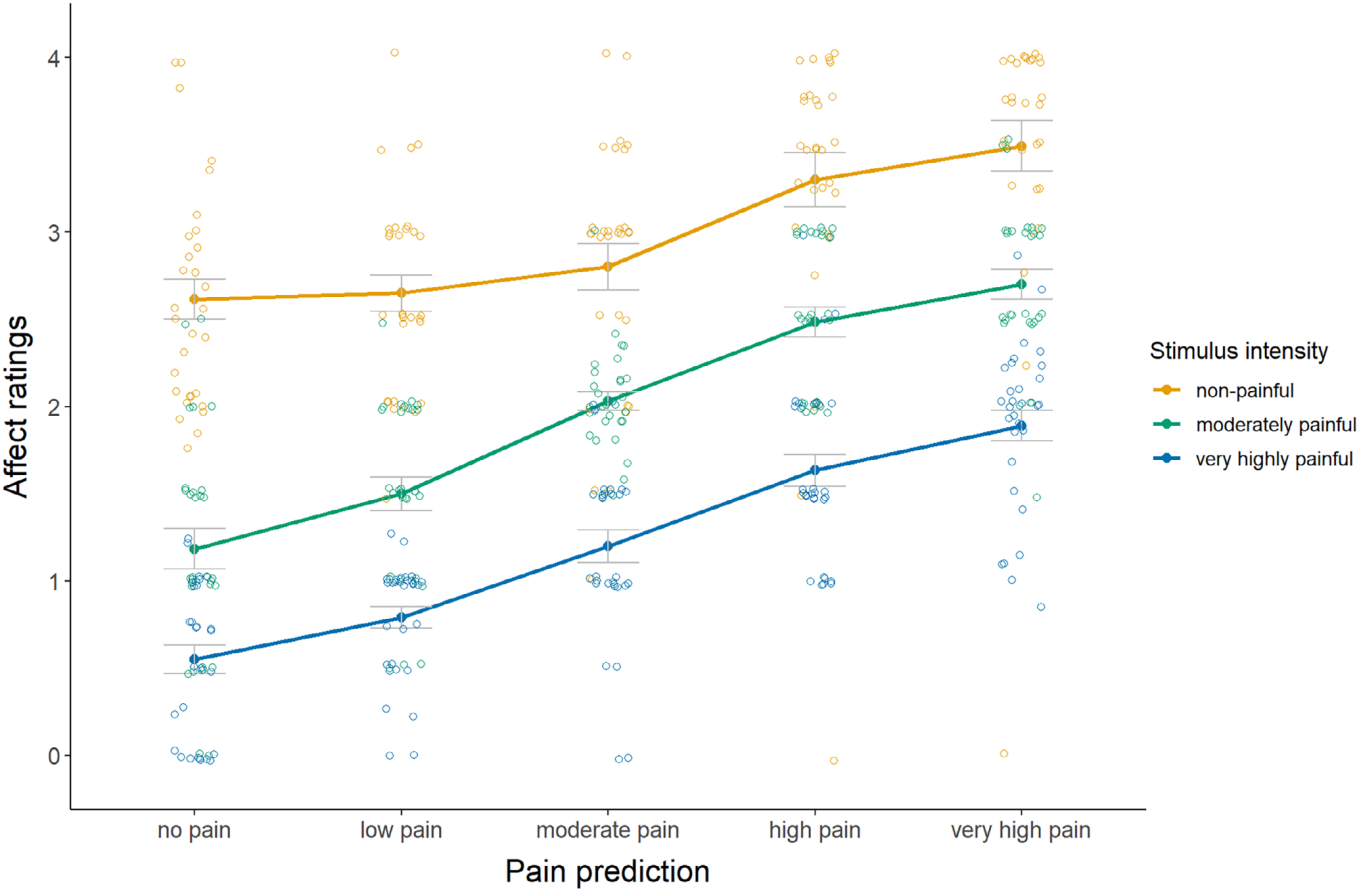
Mean affective response ratings (± standard error) for each cue and stimulus intensity (study 1). Affective responses to experiencing the predicted pain stimuli were measured on a 5‐point Likert scale ranging from 0 = very disappointed, 1 = disappointed, 2 = neutral, 3 = relieved, to 4 = very relieved.

For the secondary hypothesis (see Figure 3; Table S3 for descriptives), results indicated a significant interaction effect between pain prediction and stimulus intensity on affective responses, *F*(4.27, 123.72) = 2.91, *p* = 0.022, *η*_*g*_^2^ = 0.04. Follow‐up pairwise comparisons (see Table S5 for full test results) of the non‐painful stimulus indicated significantly stronger relief upon large and very large overprediction (PE = 6, *d* = 0.61; PE = 8, *d* = 0.81) as compared to correct prediction, while small and moderate overprediction did not yield significantly different relief (PE = 2, *d* = 0.05; PE = 4, *d* = 0.20). For the moderately painful stimulus, results indicated significant relief upon both small and moderate overprediction (PE = 2, *d* = 1.11; PE = 4, *d* = 1.41) and significant disappointment upon both small and moderate underprediction (PE = −2, *d* = 1.21; PE = −4, *d* = 1.44). For the very highly painful stimulus, results indicated significantly larger disappointment upon all degrees of underprediction (PE = −2, *d* = 0.50; PE = −4, *d* = 1.09; PE = −6, *d* = 2.02; PE = −8, *d* = 2.14). These findings suggest that underpredictions generally resulted in disappointment, while overpredictions resulted in relief. Notably, non‐painful stimuli were associated with relief and very highly painful stimuli with disappointment, regardless of predictions given (Figure 3).

#### 3.1.4 Moderation

The effects of the pain predictions on pain intensity ratings were not significantly moderated by any of the psychological questionnaire scores, that is, trait anxiety, state anxiety, pain vigilance and awareness, interoceptive awareness, and optimism (see Table S6 for descriptives, Cronbach's alpha, and main test results). Not considering adjustments for multiple testing, there was a significant three‐way moderation between optimism (LOT‐R), the pain predictions, and stimulus intensity (*F*(5.27, 147.51) = 2.32, *p* = 0.043, *η*_*g*_^2^ = 0.01), but the effect was very small and two‐way interactions between optimism and pain prediction (*F*(3.06, 85.71) = 0.53, *p* = 669, *η*_*g*_^2^ ≤ 0.01) or stimulus intensity (*F*(1.65, 46.18) = 0.15, *p* = 0.825, *η*_*g*_^2^ ≤ 0.01) were not significant. Further, the effect of the pain predictions on pain intensity rating was not significantly moderated by variability in pain intensity ratings. Post‐hoc analyses indicated that the effects of the pain predictions on pain intensity ratings were also not significantly moderated by the degree to which participants considered the cues to be informative (Table S6).

### 3.2 Results study 2

#### 3.2.1 Participants

Two participants were excluded during screening and three participants were excluded due to unsuccessful pain calibration. All other 30 participants completed their participation in the study (76.7% female, 23.4% male; M age = 21.4, SD = 3.5). For the exit question, data from 1 participant was missing. Participants varied in the extent to which they considered the cues to be informative predictors of the electrical stimuli (*n* = 5 not at all, 7 slightly, 11 moderately, 6 very) (Table S7).

#### 3.2.2 Manipulation checks

**FIGURE 4:**
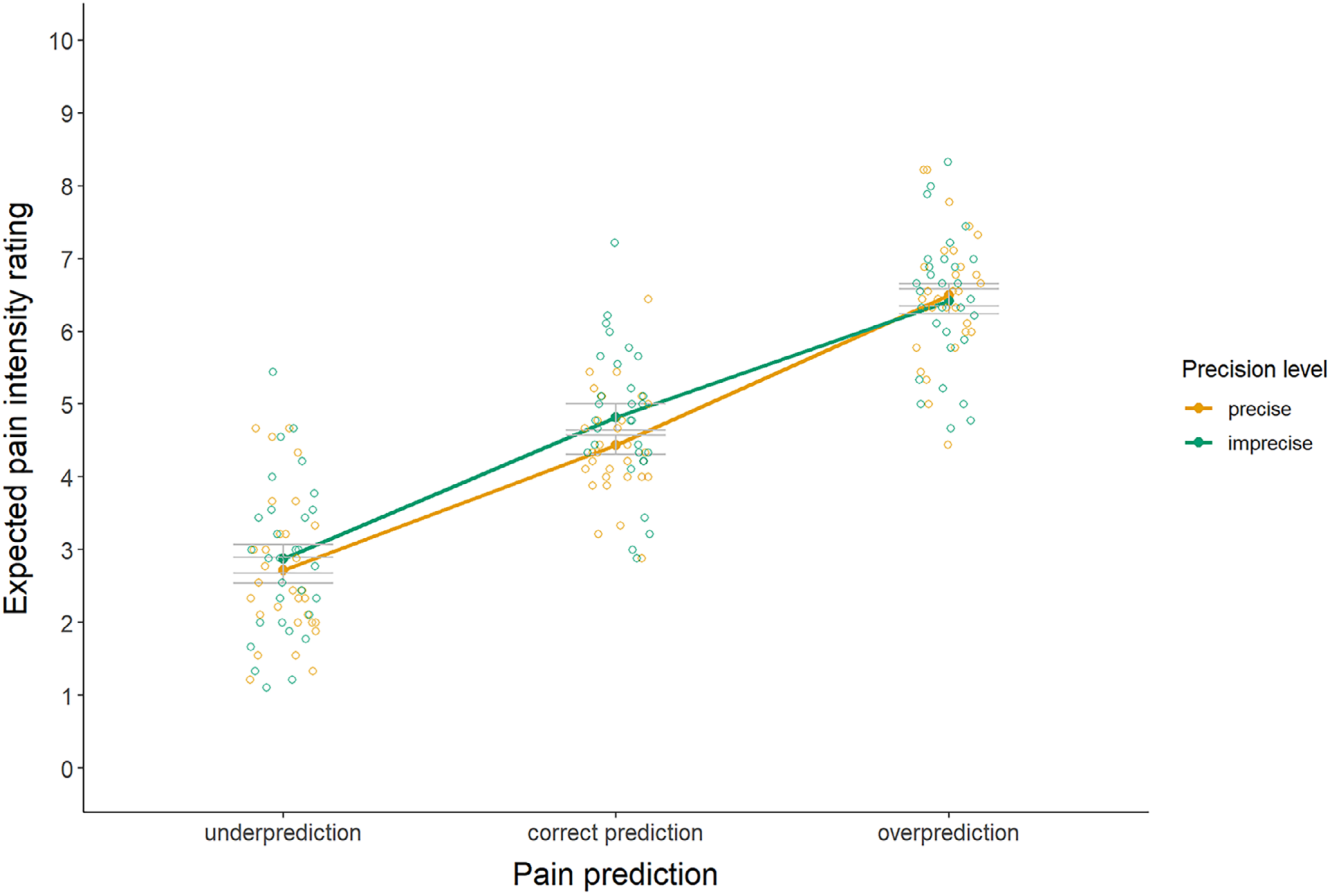
Mean pain expectation ratings (± standard error) for imprecise and precise pain predictions that provide under‐, correct, and overpredictions (study 2). Pain expectations were rated on an 11‐point numerical rating scale, where 0 means no pain at all, 2 means low pain, 4 means moderate pain, 6 means high pain, 8 means very high pain, and 10 means the most intense pain imaginable.

For the manipulation check of pain expectation ratings (see Figure 4 and Table S8 for descriptives), results indicated a significant interaction effect between precision level and magnitude of the prediction on pain expectation ratings, *F*(2, 58) = 4.29, *p* = 0.018, *η*_*g*_^2^ = 0.01. As intended, follow‐up pairwise comparisons (see Table S13 for full test results) for the imprecise pain predictions indicated significantly lower pain expectations upon underprediction versus correct prediction (*d* = 2.79), as well as higher pain expectations upon overprediction versus correct prediction (*d* = 1.84). Also, for the precise pain predictions, underpredictions resulted in significantly lower (*d* = 2.44) and overpredictions resulted in significantly higher (*d* = 2.60) pain expectations than correct predictions. Furthermore, imprecise correct predictions yielded higher expected pain than precise correct predictions (*d* = 0.58). Pain expectation ratings for imprecise versus precise predictions did not significantly differ in case of underpredictions (*d* = 0.29) or overpredictions (*d* = 0.13). These findings suggest that expectations were successfully manipulated by the magnitude of the prediction, while they were only minimally affected by the precision of the prediction.

**FIGURE 5:**
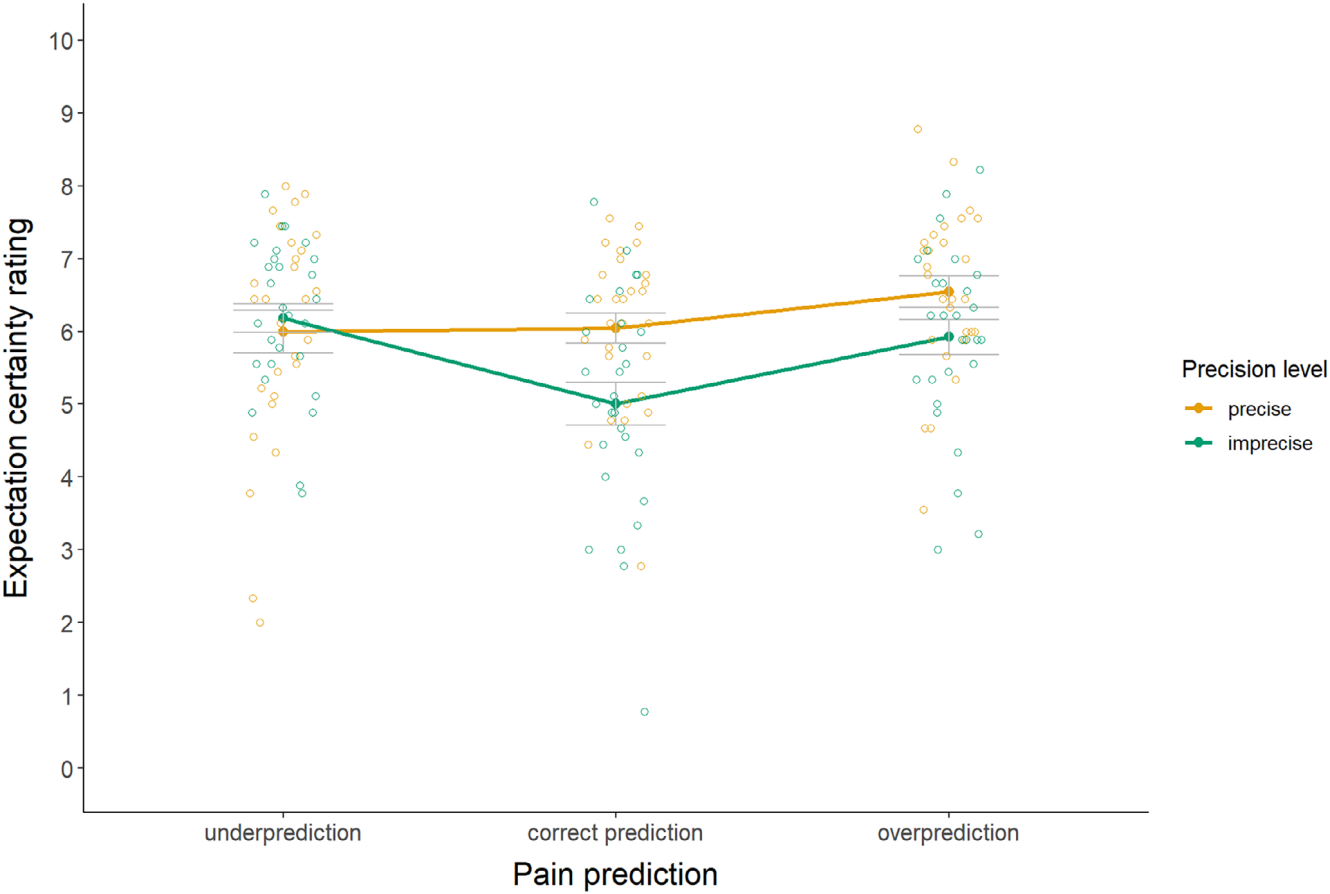
Mean certainty of pain expectation ratings (± standard error) for imprecise and precise pain predictions that provide under‐, correct, and overpredictions (study 2). Certainty of pain expectations was rated on an 11‐point numerical rating scale, where 0 means not certain at all and 10 means very certain.

Certainty of pain expectation ratings were missing for 2 participants due to a technical error. For the manipulation check of certainty (see Figure 5 and Table S9), results indicated a significant interaction effect between precision level and magnitude of the prediction, *F*(1.58, 42.69) = 11.86, *p* ≤ 0.001, *η*_*g*_^2^ = 0.04. Against our expectations, follow‐up pairwise comparisons (see Table S14 for full test results) for the imprecise pain predictions indicated significantly lower certainty of pain expectations upon underprediction than correct prediction (*d* = 0.79), as well as higher certainty of pain expectations upon overprediction than correct prediction (*d* = 0.74). For the precise pain predictions, underpredictions did not result in significantly differential certainty of pain expectations (*d* = 0.05), while overpredictions resulted in significantly higher certainty of pain expectations (*d* = 0.57) than correct predictions. Furthermore, as intended, imprecise correct predictions and imprecise overpredictions yielded lower certainty of pain expectations than precise correct or overpredictions (*d* = 0.66 and d = 0.59, respectively). However, certainty of pain expectations did not significantly differ for imprecise versus precise underpredictions (*d* = 0.19). These findings suggest that certainty was successfully manipulated only for correct and overpredictions of pain, while it was also influenced by the magnitude of predictions.

#### 3.2.3 Effect of precision and magnitude of pain prediction on pain intensity rating

**FIGURE 6:**
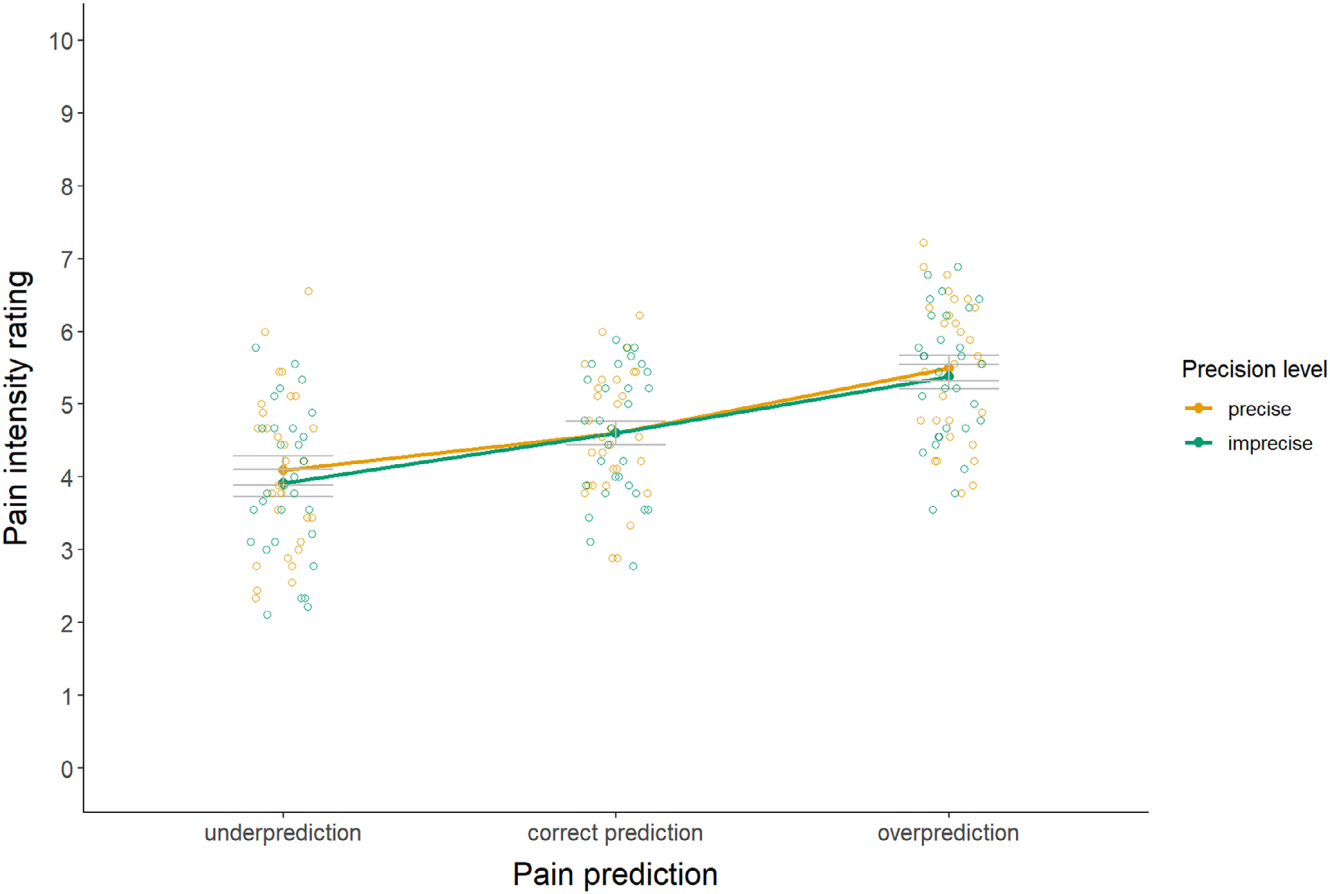
Mean pain intensity ratings (± standard error) for imprecise and precise pain predictions that provide under‐, correct, and overpredictions (study 2). Pain intensity was rated on an 11‐point numerical rating scale, where 0 means no pain at all, 2 means low pain, 4 means moderate pain, 6 means high pain, 8 means very high pain, and 10 means the most intense pain imaginable.

For the primary hypothesis (see Figure 6 and Table S10 for descriptives), results indicated no significant interaction effect between precision level and magnitude of the prediction on pain intensity ratings, *F*(2, 58) = 1.28, *p* = 0.287, *η*_*g*_^2^ ≤ 0.01, nor a main effect of precision level, *F*(1, 29) = 3.70, *p* = 0.064, *η*_*g*_^2^ ≤ 0.01. There was a significant main effect of the magnitude of the prediction on pain intensity ratings, *F*(1.26, 36.47) = 94.55, *p* ≤ 0.001, *η*_*g*_^2^ = 0.28. Follow‐up pairwise comparisons (see Table S15 for full test results) disregarding precision level indicated significant assimilation of pain ratings upon underprediction versus correct prediction (*d* = 1.43) and upon overprediction versus correct prediction (*d* = 1.73). These findings suggest that only the magnitude of predictions, but not the precision level, affect pain ratings.

Post‐hoc analyses examining possible time effects by examining the pain ratings averaged separately for each of the three blocks of the cued pain task indicated similar results as for the ratings averaged across blocks, with no clear differential effects over time (first block: interaction effect *F*(2, 58) = 0.47 *p* = 0.625, *η*_*g*_^2^ ≤ 0.01, main effect precision *F*(1, 29) = 2.40, *p* = 0.132, *η*_*g*_^2^ ≤ 0.01, main effect magnitude *F*(1.31, 37.95) = 59.15, *p* ≤ 0.001, *η*_*g*_^2^ = 0.18; second block: interaction effect *F*(2, 58) = 1.18, *p* = 0.315, η_g_^2^ ≤ 0.01, main effect precision *F*(1, 29) = 0.54, *p* = 0.469, *η*_*g*_^2^ ≤ 0.01, main effect magnitude *F*(1.51, 43.65) = 55.09, *p* ≤ 0.001, *η*_*g*_^2^ = 0.20; third block: interaction effect *F*(2, 58) = 0.02, *p* = 0.982, *η*_*g*_^2^ ≤ 0.01, main effect precision *F*(1, 29) = 0.53, *p* = 0.473, *η*_*g*_^2^ ≤ 0.01, main effect magnitude *F*(1.46, 42.33) = 68.36, *p* ≤ 0.001, *η*_*g*_^2^ = 0.23). See Table S10 for descriptives and Table S15 for full test results pairwise comparisons.

#### 3.2.4 Effect of precision and magnitude of pain prediction on affective response

**FIGURE 7:**
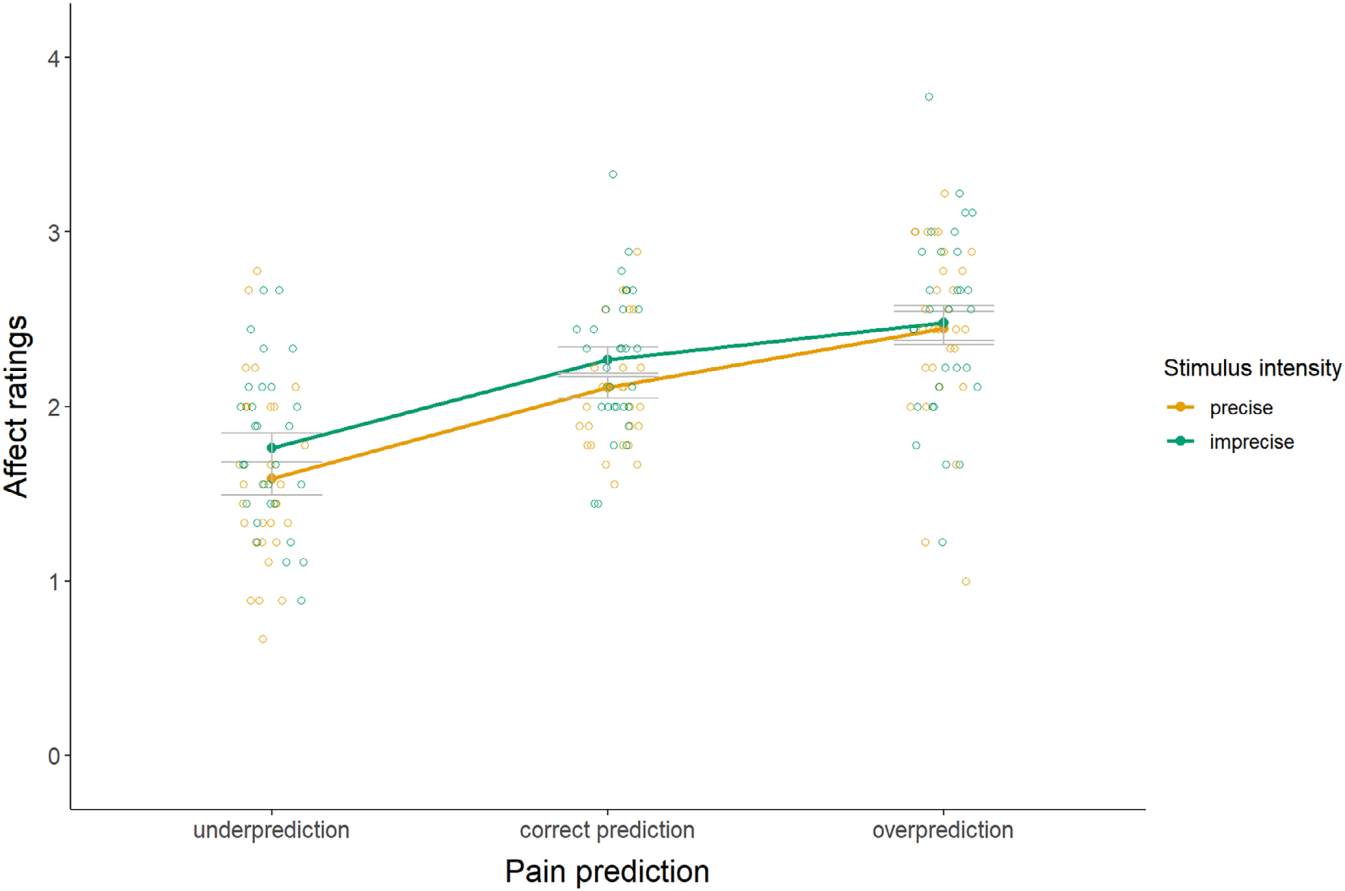
Mean affective response ratings (± standard error) for imprecise and precise pain predictions that provide under‐, correct, and overpredictions (study 2). Affective responses to experiencing the predicted pain stimuli were measured on a 5‐point Likert scale ranging from 0 = very disappointed, 1 = disappointed, 2 = neutral, 3 = relieved, to 4 = very relieved.

For the secondary hypothesis (see Figure 7 and Table S11 for descriptives), results revealed no significant interaction effect between precision level and magnitude of the pain prediction on affective responses, *F*(2, 58) = 1.47 *p* = 0.239, *η*_*g*_^2^ ≤ 0.01. There was a main effect of precision level *F*(1, 29) = 11.55 *p* = 0.002, *η*_*g*_^2^ = 0.02, indicating higher disappointment upon precise predictions than imprecise predictions. There was also a main effect of magnitude of the pain prediction on affective responses *F*(1.28, 37.01) = 34.84 *p* ≤ 0.001, *η*_*g*_^2^ = 0.33. Follow‐up pairwise comparisons (see Table S16 for full test results) disregarding precision level indicated significant disappointment upon underprediction as compared to correct prediction (*d* = 1.20) and significant relief upon overprediction as compared to correct prediction (*d* = 0.69). These findings suggest that overpredictions result in relief, while underpredictions result in disappointment, especially when the predictions are precise.

#### 3.2.5 EMG

**FIGURE 8:**
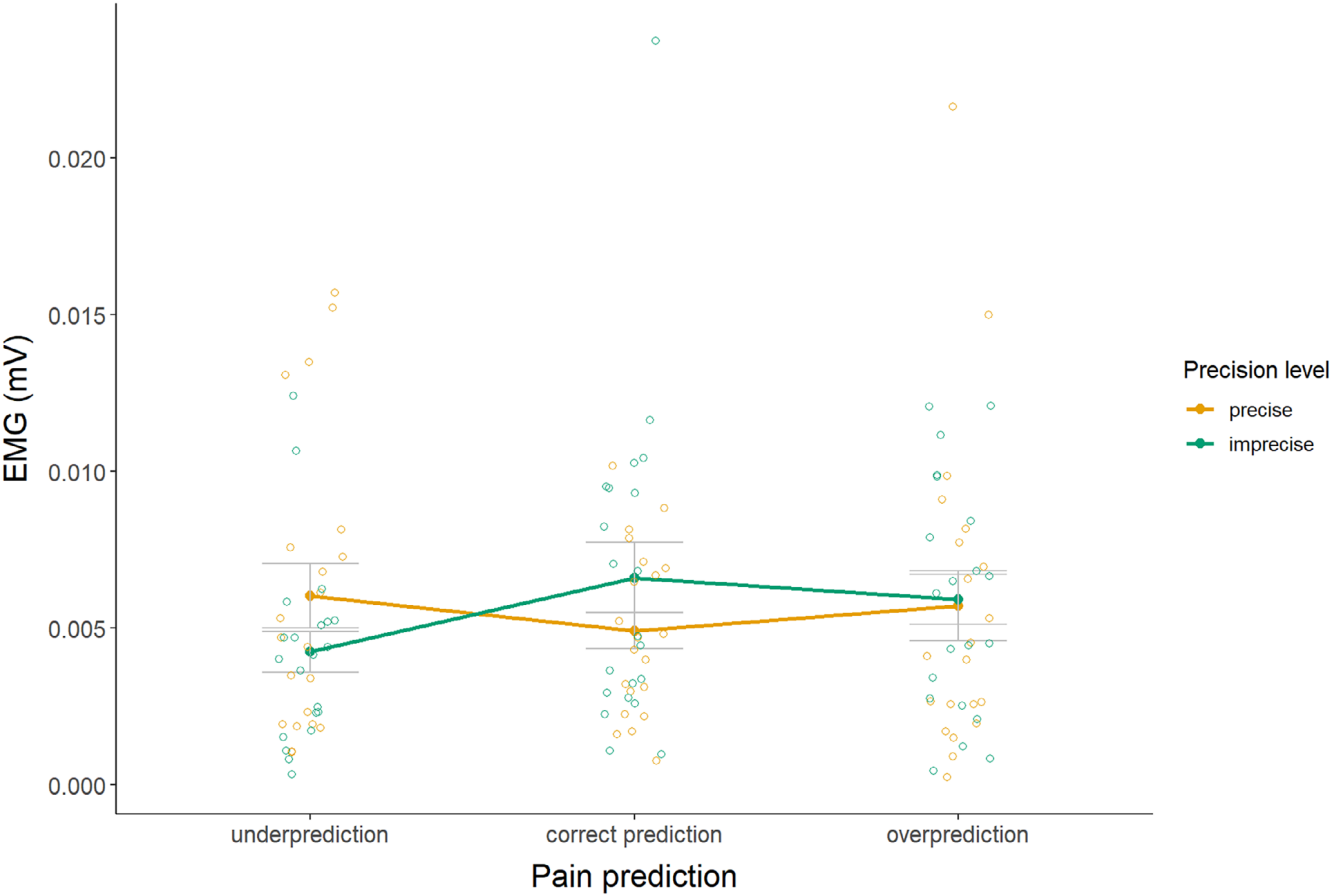
Mean peak amplitude EMG responses in mV (± standard error) for imprecise and precise pain predictions that provide under‐, correct, and overpredictions (study 2).

The EMG recordings of 9 participants were faulty due to technical difficulties and were excluded from the analyses. In addition, about 20% of trials were marked as reject trials due to noisy baseline. Results (see Figure 8 and Table S12 for descriptives) revealed no significant interaction effect between precision level and magnitude of the pain prediction on EMG eyeblink startle responses, *F*(2, 40) = 2.288, *p* = 0.115, *η*_*g*_^2^ = 0.03. Also, the main effects of precision level of the pain prediction, *F*(1, 20) = 0.003, *p* = 0.954, *η*_*g*_^2^ ≤ 0.01, and of magnitude of the pain prediction on EMG eyeblink startle responses were not significant, *F*(2, 40) = 0.69, *p* = 0.507, *η*_*g*_^2^ = 0.01. These findings suggest that magnitude and level of precision of predictions did not affect startle responses.

#### 3.2.6 Moderation

The effects of the pain predictions on pain intensity rating were not significantly moderated by any of the psychological questionnaire scores, that is, trait anxiety, state anxiety, pain vigilance and awareness, intolerance of uncertainty, and optimism (see Table S17 for descriptives, Cronbach's alpha, and main test results). There was also no significant 3‐way interaction of the magnitude and precision level of the predictions with the variability in pain intensity ratings, nor a 2‐way interaction of magnitude of the predictions with the variability of pain ratings. Not considering adjustments for multiple testing, there was a 2‐way interaction of precision level of the predictions with the variability ratings (*F*(1,28) = 5.98, *p* = 0.021, *η*_*g*_^2^ ≤ 0.01). However, follow‐up analyses did not indicate significant correlations between the variability and pain ratings for either the precise or imprecise predictions (*r* = 0.14, *p* = 0.451; *r* = 0.02, *p* = 935, respectively). Post‐hoc analyses indicated that the effects of the pain predictions on pain intensity ratings were not significantly moderated by the degree to which participants considered the cues to be informative (Table S17).

## 4 DISCUSSION

In two experimental studies, we investigated the effects of the magnitude and precision of pain predictions on pain experiences and affective responses of disappointment and relief. We found assimilation of pain experiences towards over‐ and underpredictions of pain, whereby greater prediction errors did not consistently result in greater assimilation effects. Imprecise predictions did not significantly reduce assimilation compared to precise predictions. Moreover, overprediction of pain tended to result in affective relief, while underpredictions resulted in disappointment. We did not observe an effect of the magnitude and precision of pain expectations on eyeblink startle responses. We found no indications for individual differences in several psychological characteristics and pain rating variability to moderate the effects of the predictions on pain.

The observed assimilation of pain experiences in both studies 1 and 2 is in line with the literature on expectancy effects in pain suggesting that, in general, expectations work in a self‐confirming manner (Peerdeman et al., 2016). However, assimilation was not consistently larger when the discrepancy between pain prediction and pain sensation were larger, as simple instantiations of Bayesian models including predictive processing suggest (Büchel et al., 2014; Ongaro & Kaptchuk, 2019; Tabor & Burr, 2019). This finding is in line with recent studies where boundary effects were also observed, with the largest prediction errors not resulting in the strongest assimilation effects (Hird et al., 2019; Peerdeman et al., 2021). The mechanisms for boundary effects are to be investigated further. One possibility is that the large discrepancies cause rapid re‐evaluation of the reliability of cues (Yu et al., 2009). Various participants considered the cues to be not or only slightly informative post‐test and they might not have let these cues affect their experiences when prediction errors were large. Also, the high precision of the stimuli (i.e. likelihood) might override the cues particularly when prediction errors are large. Moreover, our study suggests that overpredictions of pain may not or only slightly affect experiences of non‐painful stimuli. This may relate to peripheral or spinal processes, including different mechanisms that underlie hyperalgesia (i.e. increased pain during painful stimuli) and allodynia (experience of pain during normally non‐painful stimuli) (Sandkühler, 2009), both of which are different types of ‘learning’ process. Notably, pain experiences during the moderately painful stimuli do not suggest differential sizes of the effects of under‐ versus overpredictions on pain (note that these comparisons could not be made for the non‐painful and very highly painful stimuli as these could not be underpredicted or overpredicted, respectively). In sum, our and recent findings might indicate limitations of a simple implementation of Bayesian models by suggesting that a larger magnitude of expectations (i.e. larger prediction errors) does not reliably result in greater assimilation of pain experiences. Additional research is warranted for replication of these findings and further insight into the underlying mechanisms. One possibility is that additional factors obscure to some extent the aspects of prediction being tested here, such as participants employing trial‐by‐trial updating of their judgement of the cues (i.e. the cues may not act as static, stable predictors, but instead are updated through experience during the task), or hidden order effects, differences in the interpretation of linguistic descriptors, the potential for participants to build a variety of internal cognitive models of the experiment that distort their predictions, and various other adaptive processes, both peripheral and central. This illustrates a limitation in task designs such as this, as it is not simple to quantitatively capture all factors potentially involved.

In study 2, we manipulated the precision of pain predictions by indicating a range of pain intensity to be expected and did not find, in contrast to our predictions based on Bayesian models (Büchel et al., 2014; Ongaro & Kaptchuk, 2019; Tabor & Burr, 2019), that imprecise predictions reduced assimilation. This finding is in line with a recent meta‐analysis that did not find an influence of uncertainty on pain (Pavy, Zaman, Van Den Noortgate, et al., 2024). Note, however, that manipulation checks indicated that while expectations were successfully manipulated by the magnitude of both precise and imprecise predictions (despite imprecise predictions also including a correct prediction), certainty appeared only successfully manipulated for correct and overpredictions of pain and was influenced by the magnitude of predictions. Moreover, post‐test, various participants considered the cues to be not or only slightly informative. Further comparative research might give insight into how uncertainty can be manipulated more effectively to get a better understanding of how uncertainty then affects pain experiences. For example, a recent meta‐analysis did not indicate an influence of type of unpredictability (e.g. regarding intensity or onset), but did suggest effects may depend on targeted stimulus pain intensity, expected pain intensity, and state negative affectivity (Pavy, Zaman, Van Den Noortgate, et al., 2024). These findings rationalize an appeal to more quantitative approaches, which capture the multiple types of uncertainty that are at play and allow more formal model testing and comparison, exploiting in particular the sorts of trial‐by‐trial effects that can operate in various types of task paradigm as specific predictions of Bayesian models.

Both studies showed that underpredictions of pain led to disappointment, while overpredictions generally led to affective relief. These effects were increasingly large with increasing prediction errors (study 1), and disappointment was higher upon precise than imprecise predictions (study 2). Our findings are in line with previous studies suggesting that underpredictions can cause pain relief, but also undesirable outcomes like disappointment and trust violation (Campbell & Guy, 2007; Herruer et al., 2015; Husain & Lee, 2015; Peerdeman et al., 2021). While relief upon pain overprediction may be considered beneficial, our study, like previous research (Thomaidou et al., 2023), shows that overprediction can also intensify pain. Moreover, expectations of high pain have been associated with increased levels of fear and anxiety (Benedetti et al., 2007). In study 2, we did, however, not find that the disconfirmation of precise or imprecise under‐ or overpredictions of pain significantly influenced EMG eyeblink startle responses, an indicator of pain‐related fear (van Boxtel, 2010). Similar patterns of EMG eyeblink startle responses have been found in studies that also conduct tasks where pain is directly targeted (Pinkney et al., 2014). In sum, our findings underline the importance of providing correct, realistic predictions of upcoming pain in order to balance optimal pain and affective responses.

Our studies did not indicate any moderation of the effect of pain predictions on pain experiences by the psychological characteristics studied (i.e. trait anxiety, state anxiety, pain vigilance and awareness, interoceptive awareness, intolerance of uncertainty, and optimism). This finding is largely in line with the existing literature, in which psychological characteristics are generally not found to consistently predict expectancy effects (Horing et al., 2014; Kang et al., 2023). These null findings might partially be due to the small sample size in the current and previous studies, since associations with psychological characteristics may generally be small. However, we should also consider that expectancy effects on pain may not be reliably related to stable psychological characteristics and are determined by interactions between different factors, including the context. A potentially relevant predictor that has only recently been studied, is variability of pain ratings. Recent research has found that individuals who were more inwardly directed showed more reliable (i.e. less variable) pain reporting and were less influenced by external pain predictions than externally directed individuals (Treister et al., 2019). In the current studies, we did not find variability of pain ratings during an uncued pain task to moderate the effects of the pain predictions on pain experiences. This might be related to a different, simpler approach of calculating the variability in pain reporting compared to previous studies (Treister et al., 2017). Finally, also the degree to which participants considered the cues to be informative did not significantly moderate the effects.

When considering the implications of the current findings, the limitations of our research should be considered. First, these are experimental lab studies. Although this allows for control and safety, patients do not have the reassurance that the pain they are about to experience is within a certain limit and without lasting effects, nor can it always be predicted. Hence, anxiety, uncertainty, and expectations of higher pain are likely increased in clinical settings. Second, pain experiences are not only influenced by a single pain prediction made just prior to receiving a painful stimulus. Pain experiences can be influenced by many factors, including previous experiences, such as the predictions and stimuli during preceding trials in this study (Jepma et al., 2018; Pavy, Zaman, Von Leupoldt, & Torta, 2024), anxiety about or fear of the painful procedure, and attentional focus (Bushnell et al., 2013; Pagnini et al., 2023). Third, the majority of our sample was female (87% in study 1; 77% in study 2). Previous research indicates sex differences in pain experiences (Mogil, 2020) and women have been found to be less sensitive than men to placebo effects (of which expectancies are a core mechanism) evoked through verbal suggestions (Enck & Klosterhalfen, 2019; Vambheim & Flaten, 2017). With these limitations in mind, future research could more closely resemble clinical practice. Another limitation is that, although neurobiological mechanisms have been found to underlie expectancy effects (Schedlowski et al., 2015), response biases cannot be fully excluded in the current studies. Also, inclusion of larger sample sizes for enhanced statistical power, variation of the prior‐to‐likelihood ratio (i.e. the strength of the prediction versus the stimulus), and applying computational models to the data might allow more fine‐grained insights into how well Bayesian models inform on the way that pain predictions shape pain experiences (Büchel et al., 2014; Ongaro & Kaptchuk, 2019; Pagnini et al., 2023; Tabor & Burr, 2019).

In conclusion, we found that the magnitude of pain predictions influenced pain experiences, with under‐ and overpredictions of pain causing assimilation of pain experiences. However, larger prediction errors did not consistently result in greater assimilation. Moreover, underpredictions generally resulted in disappointment, while overpredictions resulted in affective relief. We did not find precise and imprecise pain predictions to influence pain experiences differentially, though precise predictions did yield slightly greater disappointment than imprecise predictions. Our findings indicate that healthcare professionals should be careful when communicating predictions about upcoming painful procedures. Even though pain relief can be accomplished through underprediction of pain, patients may experience adverse emotions, such as disappointment. Implications might extend to acute and chronic pain. This may be particularly relevant when considering that frequent heightened pain expectations may contribute to the persistence of pain (Büchel, 2023). Possibly, also the violation of expectations of pain relief (i.e. underpredictions) may negatively affect pain persistence given the observed adverse emotional effects. Thus, our findings underline the importance of establishing correct expectations to improve patient outcomes.

## AUTHOR CONTRIBUTIONS

**Suzanne Derksen**: conceptualization, methodology, software, investigation, data curation, writing—review & editing, supervision, project administration; **Maria Konttinen**: conceptualization, investigation, review & editing; **Anastasiia Myronenko**: conceptualization, investigation, review & editing; **Ben Seymour**: conceptualization, review & editing; **Kaya Peerdeman**: conceptualization, methodology, validation, data curation, formal analysis, writing—review & editing, visualization, supervision, project administration, funding acquisition.

## CONFLICT OF INTEREST STATEMENT

The authors have nothing to report.

## Supporting information


Data S1.

## References

[ejp4769-bib-0001] Arntz, A. , & Hopmans, M. (1998). Underpredicted pain disrupts more than correctly predicted pain, but does not hurt more. Behaviour Research and Therapy, 36(12), 1121–1129. 10.1016/S0005-7967(98)00085-0 9745797

[ejp4769-bib-0002] Arntz, A. , van den Hout, M. A. , van den Berg, G. , & Meijboom, A. (1991). The effects of incorrect pain expectations on acquired fear and pain responses. Behaviour Research and Therapy, 29(6), 547–560. 10.1016/0005-7967(91)90005-N 1759955

[ejp4769-bib-0003] Aslaksen, P. M. , Åsli, O. , Øvervoll, M. , & Bjørkedal, E. (2016). Nocebo hyperalgesia and the startle response. Neuroscience, 339, 599–607. 10.1016/j.neuroscience.2016.10.040 27789385

[ejp4769-bib-0004] Benedetti, F. , Lanotte, M. , Lopiano, L. , & Colloca, L. (2007). When words are painful: Unraveling the mechanisms of the nocebo effect. In NEUROSCIENCE (Vol. 147, pp. 260–271). PERGAMON‐ELSEVIER SCIENCE LTD. . 10.1016/j.neuroscience.2007.02.020 17379417

[ejp4769-bib-0005] Blumenthal, T. D. , Cuthbert, B. N. , Filion, D. L. , Hackley, S. , Lipp, O. V. , & Van Boxtel, A. (2005). Committee report: Guidelines for human startle eyeblink electromyographic studies. Psychophysiology, 42(1), 1–15. 10.1111/j.1469-8986.2005.00271.x 15720576

[ejp4769-bib-0006] Blythe, J. S. , Thomaidou, M. A. , Peerdeman, K. J. , van Laarhoven, A. I. M. , Van Schothorst, M. M. E. , Veldhuijzen, D. S. , & Evers, A. W. M. (2022). Placebo effects on cutaneous pain and itch: A systematic review and meta‐analysis of experimental results and methodology. Pain, 164, 1181–1199. 10.1097/j.pain.0000000000002820 36718994 PMC10184563

[ejp4769-bib-0007] Büchel, C. (2023). The role of expectations, control and reward in the development of pain persistence based on a unified model. eLife, 12, e81795. 10.7554/eLife.81795 36972108 PMC10042542

[ejp4769-bib-0008] Büchel, C. , Geuter, S. , Sprenger, C. , & Eippert, F. (2014). Placebo analgesia: A predictive coding perspective. Neuron, 81(6), 1223–1239. 10.1016/j.neuron.2014.02.042 24656247

[ejp4769-bib-0009] Bushnell, M. C. , Čeko, M. , & Low, L. A. (2013). Cognitive and emotional control of pain and its disruption in chronic pain. Nature Reviews Neuroscience, 14(7), 502–511. 10.1038/nrn3516 23719569 PMC4465351

[ejp4769-bib-0010] Campbell, C. , & Guy, A. (2007). `why Can't they do anything for a simple Back problem?’: A qualitative examination of expectations for Low Back pain treatment and outcome. Journal of Health Psychology, 12(4), 641–652. 10.1177/1359105307078171 17584815

[ejp4769-bib-0011] Carleton, R. N. , & Asmundson, G. J. G. (2007). Fearing the unknown: A short version of the intolerance of uncertainty scale. Journal of Anxiety Disorders, 14, 105–117. 10.1016/j.janxdis.2006.03.014 16647833

[ejp4769-bib-0012] Cohen, J. (1988). Statistical power analysis for the behavioral sciences ((2nd ed). ed.). L. Erlbaum Associates.

[ejp4769-bib-0055] Enck, P. , & Klosterhalfen, S. (2019). Does sex/gender play a role in placebo and nocebo effects? Conflicting evidence from clinical trials and experimental studies. Frontiers in Neuroscience, 13, 160. 10.3389/fnins.2019.00160 30886569 PMC6409330

[ejp4769-bib-0013] Evers, A. W. M. , Colloca, L. , Blease, C. , Annoni, M. , Atlas, L. Y. , Benedetti, F. , Bingel, U. , Büchel, C. , Carvalho, C. , Colagiuri, B. , Crum, A. J. , Enck, P. , Gaab, J. , Geers, A. L. , Howick, J. , Jensen, K. B. , Kirsch, I. , Meissner, K. , Napadow, V. , … Kelley, J. (2018). Implications of placebo and nocebo effects for clinical practice: Expert consensus. Psychotherapy and Psychosomatics, 87(4), 204–210. 10.1159/000490354 29895014 PMC6191882

[ejp4769-bib-0014] Evers, A. W. M. , Colloca, L. , Blease, C. , Gaab, J. , Jensen, K. B. , Atlas, L. Y. , Beedie, C. J. , Benedetti, F. , Bingel, U. , Büchel, C. , Bussemaker, J. , Colagiuri, B. , Crum, A. J. , Finniss, D. G. , Geers, A. L. , Howick, J. , Klinger, R. , Meeuwis, S. H. , Meissner, K. , … Kirsch, I. (2021). What should clinicians tell patients about placebo and nocebo effects? Practical considerations based on expert consensus. Psychotherapy and Psychosomatics, 90(1), 49–56. 10.1159/000510738 33075796

[ejp4769-bib-0015] Fazeli, S. , & Büchel, C. (2018). Pain‐related expectation and prediction error signals in the anterior insula are not related to aversiveness. The Journal of Neuroscience, 38(29), 6461–6474. 10.1523/JNEUROSCI.0671-18.2018 29934355 PMC6705956

[ejp4769-bib-0016] Field, A. P. , Miles, J. , & Field, Z. (2012). Discovering statistics using R. Sage. http://catalog.hathitrust.org/api/volumes/oclc/760970657.html.

[ejp4769-bib-0017] Geuter, S. , Boll, S. , Eippert, F. , & Büchel, C. (2017). Functional dissociation of stimulus intensity encoding and predictive coding of pain in the insula. eLife, 6, e24770. 10.7554/eLife.24770 28524817 PMC5470871

[ejp4769-bib-0018] Grahl, A. , Onat, S. , & Büchel, C. (2018). The periaqueductal gray and Bayesian integration in placebo analgesia. eLife, 7, e32930. 10.7554/eLife.32930 29555019 PMC5860873

[ejp4769-bib-0019] Herruer, J. M. , Prins, J. B. , van Heerbeek, N. , Verhage‐Damen, G. W. J. A. , & Ingels, K. J. A. O. (2015). Negative predictors for satisfaction in patients seeking facial cosmetic surgery: A systematic review. Plastic and Reconstructive Surgery, 135(6), 1596–1605. 10.1097/PRS.0000000000001264 26017596

[ejp4769-bib-0020] Hird, E. J. , Charalambous, C. , El‐Deredy, W. , Jones, A. K. , & Talmi, D. (2019). Boundary effects of expectation in human pain perception. Scientific Reports, 9(1), 9443. 10.1038/s41598-019-45811-x 31263144 PMC6602973

[ejp4769-bib-0021] Horing, B. , Weimer, K. , Muth, E. R. , & Enck, P. (2014). Prediction of placebo responses: A systematic review of the literature. Frontiers in Psychology, 5, 1079. 10.3389/fpsyg.2014.01079 25324797 PMC4181242

[ejp4769-bib-0022] Hoskin, R. , Berzuini, C. , Acosta‐Kane, D. , El‐Deredy, W. , Guo, H. , & Talmi, D. (2019). Sensitivity to pain expectations: A Bayesian model of individual differences. Cognition, 182, 127–139. 10.1016/j.cognition.2018.08.022 30243037

[ejp4769-bib-0023] Husain, A. , & Lee, G.‐C. (2015). Establishing realistic patient expectations following Total knee arthroplasty. Journal of the American Academy of Orthopaedic Surgeons, 23(12), 707–713. 10.5435/JAAOS-D-14-00049 26493969

[ejp4769-bib-0024] Jepma, M. , Koban, L. , van Doorn, J. , Jones, M. , & Wager, T. D. (2018). Behavioural and neural evidence for self‐reinforcing expectancy effects on pain. Nature Human Behaviour, 2(11), 838–855. 10.1038/s41562-018-0455-8 PMC6768437 31558818

[ejp4769-bib-0025] Kang, H. , Miksche, M. S. , & Ellingsen, D.‐M. (2023). Association between personality traits and placebo effects: A preregistered systematic review and meta‐analysis. Pain, 164(3), 494–508. 10.1097/j.pain.0000000000002753 35947877

[ejp4769-bib-0026] Kirsch, I. (1985). Response expectancy as a determinant of experience and behavior. American Psychologist, 40(11), 1189–1202.

[ejp4769-bib-0027] Lakens, D. (2013). Calculating and reporting effect sizes to facilitate cumulative science: A practical primer for t‐tests and ANOVAs. Frontiers in Psychology, 4, 863. 24324449 10.3389/fpsyg.2013.00863 PMC3840331

[ejp4769-bib-0028] Marteau, T. M. , & Bekker, H. (1992). The development of a six‐item short‐form of the state scale of the Spielberger state–trait anxiety inventory (STAI). British Journal of Clinical Psychology, 31(3), 301–306. 10.1111/j.2044-8260.1992.tb00997.x 1393159

[ejp4769-bib-0029] Mathôt, S. , Schreij, D. , & Theeuwes, J. (2012). OpenSesame: An open‐source, graphical experiment builder for the social sciences. Behavior Research Methods, 44(2), 314–324. 10.3758/s13428-011-0168-7 22083660 PMC3356517

[ejp4769-bib-0030] Mehling, W. E. , Acree, M. , Stewart, A. , Silas, J. , & Jones, A. (2018). The multidimensional assessment of interoceptive awareness, version 2 (MAIA‐2). PLoS One, 13(12), e0208034. 10.1371/journal.pone.0208034 30513087 PMC6279042

[ejp4769-bib-0031] Mogil, J. S. (2020). Qualitative sex differences in pain processing: Emerging evidence of a biased literature. Nature Reviews Neuroscience, 21(7), 353–365. 10.1038/s41583-020-0310-6 32440016

[ejp4769-bib-0032] Ongaro, G. , & Kaptchuk, T. J. (2019). Symptom perception, placebo effects, and the Bayesian brain. Pain, 160(1), 1–4. 10.1097/j.pain.0000000000001367 30086114 PMC6319577

[ejp4769-bib-0033] Pagnini, F. , Barbiani, D. , Cavalera, C. , Volpato, E. , Grosso, F. , Minazzi, G. A. , Vailati Riboni, F. , Graziano, F. , Di Tella, S. , Manzoni, G. M. , Silveri, M. C. , Riva, G. , & Phillips, D. (2023). Placebo and nocebo effects as Bayesian‐brain phenomena: The overlooked role of likelihood and attention. Perspectives on Psychological Science, 18(5), 1217–1229. 10.1177/17456916221141383 36656800

[ejp4769-bib-0034] Pavy, F. , Zaman, J. , Van Den Noortgate, W. , Scarpa, A. , Von Leupoldt, A. , & Torta, D. M. (2024). The effect of unpredictability on the perception of pain: A systematic review and meta‐analysis. Pain, 165, 1702–1718. 10.1097/j.pain.0000000000003199 38422488

[ejp4769-bib-0035] Pavy, F. , Zaman, J. , Von Leupoldt, A. , & Torta, D. M. (2024). Expectations underlie the effects of unpredictable pain: A behavioral and electroencephalogram study. Pain, 165, 596–607. 10.1097/j.pain.0000000000003046 37703404

[ejp4769-bib-0036] Peerdeman, K. J. , Geers, A. L. , Della Porta, D. , Veldhuijzen, D. S. , & Kirsch, I. (2021). Underpredicting pain: An experimental investigation into the benefits and risks. Pain, 162(7), 2024–2035. 10.1097/j.pain.0000000000002199 33470747

[ejp4769-bib-0037] Peerdeman, K. J. , van Laarhoven, A. I. M. , Peters, M. L. , & Evers, A. W. M. (2016). An integrative review of the influence of expectancies on pain. Frontiers in Psychology, 7, 1270. 10.3389/fpsyg.2016.01270 27602013 PMC4993782

[ejp4769-bib-0038] Pinkney, V. , Wickens, R. , Bamford, S. , Baldwin, D. S. , & Garner, M. (2014). Defensive eye‐blink startle responses in a human experimental model of anxiety. Journal of Psychopharmacology, 28(9), 874–880. 10.1177/0269881114532858 24899597 PMC4876426

[ejp4769-bib-0039] Pollo, A. , Amanzio, M. , Arslanian, A. , Casadio, C. , Maggi, G. , & Benedetti, F. (2001). Response expectancies in placebo analgesia and their clinical relevance. Pain, 93(1), 77–84. 10.1016/S0304-3959(01)00296-2 11406341

[ejp4769-bib-0040] Roelofs, J. , Peters, M. L. , McCracken, L. , & Vlaeyen, J. W. S. (2003). The pain vigilance and awareness questionnaire (PVAQ): Further psychometric evaluation in fibromyalgia and other chronic pain syndromes. Pain, 101(3), 299–306. 10.1016/S0304-3959(02)00338-X 12583873

[ejp4769-bib-0041] Sandkühler, J. (2009). Models and mechanisms of hyperalgesia and allodynia. Physiological Reviews, 89(2), 707–758. 10.1152/physrev.00025.2008 19342617

[ejp4769-bib-0042] Schedlowski, M. , Enck, P. , Rief, W. , & Bingel, U. (2015). Neuro‐bio‐behavioral mechanisms of placebo and nocebo responses: Implications for clinical trials and clinical practice. Pharmacological Reviews, 67(3), 697–730. 10.1124/pr.114.009423 26126649

[ejp4769-bib-0043] Scheier, M. F. , Carver, C. S. , & Bridges, M. W. (1994). Distinguishing optimism from neuroticism (and trait anxiety, self‐mastery, and self‐esteem): A reevaluation of the life orientation test. Journal of Personality and Social Psychology, 67(6), 1063–1078. 10.1037//0022-3514.67.6.1063 7815302

[ejp4769-bib-0044] Sjak‐Shie, E. E. (2022). PhysioData toolbox (0.6.3) [computer software]. https://PhysioDataToolbox.leidenuniv.nl

[ejp4769-bib-0045] Spielberger, C. D. , Sydeman, S. J. , Owen, A. E. , & Marsh, B. J. (1999). Measuring anxiety and anger with the state–trait anxiety inventory (STAI) and the state–trait anger expression inventory (STAXI). In The use of psychological testing for treatment planning and outcomes assessment (2nd ed., pp. 993–1021). Lawrence Erlbaum Associates Publishers.

[ejp4769-bib-0046] Tabor, A. , & Burr, C. (2019). Bayesian learning models of pain: A call to action. Current Opinion in Behavioral Sciences, 26, 54–61. 10.1016/j.cobeha.2018.10.006

[ejp4769-bib-0047] Thomaidou, M. A. , Blythe, J. S. , Peerdeman, K. J. , van Laarhoven, A. I. M. , Van Schothorst, M. M. E. , Veldhuijzen, D. S. , & Evers, A. W. M. (2023). Learned nocebo effects on cutaneous sensations of pain and itch: A systematic review and meta‐analysis of experimental behavioral studies on healthy humans. Psychosomatic Medicine, 85, 308–321. 10.1097/PSY.0000000000001194 36961347 PMC10171297

[ejp4769-bib-0048] Treister, R. , Eaton, T. , Trudeau, J. J. , Elder, H. , & Katz, P. (2017). Development and preliminary validation of the focused analgesia selection test to identify accurate pain reporters. Journal of Pain Research, 10, 319–326. 10.2147/JPR.S121455 28243138 PMC5315353

[ejp4769-bib-0049] Treister, R. , Honigman, L. , Lawal, O. D. , Lanier, R. K. , & Katz, N. P. (2019). A deeper look at pain variability and its relationship with the placebo response: Results from a randomized, double‐blind, placebo‐controlled clinical trial of naproxen in osteoarthritis of the knee. Pain, 160(7), 1522–1528. 10.1097/j.pain.0000000000001538 30817436

[ejp4769-bib-0050] van Boxtel, A. (2010). Facial EMG as a tool for inferring affective states.

[ejp4769-bib-0056] Vambheim, S. M. , & Flaten, M. A. (2017). A systematic review of sex differences in the placebo and the nocebo effect. Journal of Pain Research, 10, 1831–1839. 10.2147/JPR.S134745 28831271 PMC5548268

[ejp4769-bib-0051] Weibull, W. (1951). A statistical distribution function of wide applicability. Journal of Applied Mechanics, 18(3), 293–297. 10.1115/1.4010337

[ejp4769-bib-0052] Yoshida, W. , Seymour, B. , Koltzenburg, M. , & Dolan, R. J. (2013). Uncertainty increases pain: Evidence for a novel mechanism of pain modulation involving the periaqueductal gray. Journal of Neuroscience, 33(13), 5638–5646. 10.1523/JNEUROSCI.4984-12.2013 23536078 PMC3701089

[ejp4769-bib-0053] Yu, A. J. , Dayan, P. , & Cohen, J. D. (2009). Dynamics of attentional selection under conflict: Toward a rational Bayesian account. Journal of Experimental Psychology. Human Perception and Performance, 35(3), 700–717. 10.1037/a0013553 19485686 PMC3432507

[ejp4769-bib-0054] Zaman, J. , Vanpaemel, W. , Aelbrecht, C. , Tuerlinckx, F. , & Vlaeyen, J. W. S. (2017). Biased pain reports through vicarious information: A computational approach to investigate the role of uncertainty. Cognition, 169, 54–60. 10.1016/j.cognition.2017.07.009 28825990

