# Supplementary material for: How the magnitude and precision of pain predictions shape pain experiences
Source: Eur J Pain. 2024 Dec 13;29(1):e4769. doi: 10.1002/ejp.4769 (PMC11639048; doi:10.1002/ejp.4769)
Supplement: Supplementary file 1 — Data S1. [file EJP-29-0-s001.docx]

**Supplementary material**

# Detailed results study 1

**tableS1.** Frequency of informative predictor and attention ratings per answering option in study 1.

|  | **Not at all** | **Slightly** | **Moderately** | **Very** | **Extremely** |
| --- | --- | --- | --- | --- | --- |
| Did you think the cues were an informative predictor of the intensity of the electrical stimuli? | 3 | 12 | 8 | 7 | 0 |
| How focused were you on your sensations during the stimuli? | 0 | 1 | 1 | 22 | 6 |

**tableS2.** Mean pain intensity ratings (primary outcome) and standard deviation (SD) per cue and stimulus intensity in study 1.

| **Pain prediction** | **Non-painful  stimulus** | | **Moderately painful stimulus** | | **Very highly painful stimulus** | |
| --- | --- | --- | --- | --- | --- | --- |
|  | *Mean* | *SD* | *Mean* | *SD* | *Mean* | *SD* |
| **Average across blocks *(primary)*** |  |  |  |  |  |  |
| No pain | 0.14 | 0.27 | 3.57 | 1.54 | 6.28 | 1.67 |
| Low pain | 0.27 | 0.45 | 3.33 | 1.26 | 6.09 | 1.52 |
| Moderate pain | 0.37 | 0.63 | 4.03 | 1.15 | 6.33 | 1.54 |
| High pain | 0.18 | 0.39 | 4.38 | 1.36 | 6.78 | 1.19 |
| Very high pain | 0.11 | 0.23 | 4.60 | 1.33 | 7.13 | 1.35 |
| **Average first block** |  |  |  |  |  |  |
| No pain | 0.15 | 0.33 | 3.57 | 1.91 | 6.40 | 1.57 |
| Low pain | 0.37 | 0.67 | 3.30 | 1.47 | 5.93 | 1.70 |
| Moderate pain | 0.43 | 0.82 | 4.03 | 1.14 | 6.27 | 1.74 |
| High pain | 0.27 | 0.74 | 4.60 | 1.63 | 6.77 | 1.22 |
| Very high pain | 0.15 | 0.35 | 4.80 | 1.52 | 7.17 | 1.29 |
| **Average second block** |  |  |  |  |  |  |
| No pain | 0.13 | 0.39 | 3.57 | 1.91 | 6.15 | 1.98 |
| Low pain | 0.17 | 0.46 | 3.37 | 1.77 | 6.25 | 1.70 |
| Moderate pain | 0.30 | 0.70 | 4.02 | 1.40 | 6.40 | 1.57 |
| High pain | 0.10 | 0.31 | 4.17 | 1.80 | 6.80 | 1.45 |
| Very high pain | 0.07 | 0.22 | 4.40 | 2.14 | 7.08 | 1.51 |

*Note*. Pain intensity was rated on an 11-point numerical rating scale, where 0 means no pain at all, 2 means low pain, 4 means moderate pain, 6 means high pain, 8 means very high pain, and 10 means the most intense pain imaginable.

**tableS3**. Mean affective response ratings and standard deviation (SD) per cue and stimulus intensity in study 1.

| **Pain prediction** | **Non-painful  stimulus** | | **Moderately painful stimulus** | | **Very highly painful stimulus** | |
| --- | --- | --- | --- | --- | --- | --- |
|  | *Mean* | *SD* | *Mean* | *SD* | *Mean* | *SD* |
| No pain | 2.61 | 0.63 | 1.18 | 0.64 | 0.55 | 0.44 |
| Low pain | 2.65 | 0.58 | 1.50 | 0.53 | 0.79 | 0.34 |
| Moderate pain | 2.80 | 0.73 | 2.03 | 0.30 | 1.20 | 0.52 |
| High pain | 3.30 | 0.85 | 2.48 | 0.46 | 1.63 | 0.51 |
| Very high pain | 3.49 | 0.79 | 2.70 | 0.47 | 1.89 | 0.48 |

*Note*. Affective responses to experiencing the predicted pain stimuli were measured on a 5-point Likert scale ranging from 0 = very disappointed, 1 = disappointed, 2 = neutral, 3 = relieved, to 4 = very relieved.

**tableS4.** Full test results pairwise comparisons pain intensity ratings (primary outcome) in study 1.

| **Pain prediction** | **Non-painful**  **stimulus** | **Moderately painful stimulus** | **Very highly painful stimulus** |
| --- | --- | --- | --- |
| **Average across blocks *(primary)*** | |  |  |
| No pain | - | *t(*29) = -2.77, ***p* = .010, *d* = 0.51** * $\bar{d}$ = -0.46 [-0.80 ; -0.12] | *t*(29) = -4.51, ***p* ≤ .001, *d* = 0.82** * $\bar{d}$ = -0.85 [-1.24 ; -0.47] |
| Low pain | *t*(29) = -1.92, *p* = .064, *d* = 0.35 * $\bar{d}$ = -0.13 [-0.26 ; 0.01] | *t*(29) = -4.34, ***p* ≤ .001, *d* = 0.79** * $\bar{d}$ = -0.69 [-1.02 ; -0.37] | *t*(29) = -6.79, ***p* ≤ .001, *d* = 1.24** * $\bar{d}$ = -1.04 [-1.35 ; -0.72] |
| Moderate pain | *t*(29) = -2.24, *p* = .033, *d* = 0.41 * $\bar{d}$ = -0.23 [-0.43 ; -0.02] | - | *t*(29) = -5.09, ***p* ≤ .001, *d* = 0.93** * $\bar{d}$ = -0.79 [-1.11 ; -0.48] |
| High pain | *t*(29) = -0.80, *p* = .433, *d* = 0.15 $\bar{d}$ = -0.04 [-0.15 ; 0.07] | *t*(29) = -2.33, *p* = .027, *d* = 0.42 * $\bar{d}$ = -0.36 [-0.67 ; -0.04] | *t*(29) = -2.63, *p* = .014, *d* = 0.48 * $\bar{d}$ = -0.34 [-0.61 ; -0.08] |
| Very high pain | *t*(29) = 0.92, *p* = .363, *d* = 0.17  $\bar{d}$ = 0.03 [-0.04 ; 0.11] | *t*(29) = -3.18, ***p* = .003, *d* = 0.58** * $\bar{d}$ = -0.57 [-0.94 ; -0.20] | - |
| **Average first block** | |  |  |
| No pain |  | *t*(29) = -1.98, *p* = .057, *d* = 0.36 $\bar{d}$ = -0.47 [-0.95 ; 0.01] | *t*(29) = -3.53, ***p* = .001, *d* = 0.64** $\bar{d}$ = -0.77 [-1.22 ; -0.33] |
| Low pain | *t*(29) = -2.20, *p* = .036, *d* = 0.40 $\bar{d}$ = -0.22 [-0.42 ; -0.02] | *t*(29) = -3.79, ***p* ≤ .001, *d* = 0.69** $\bar{d}$ = -0.73 [-1.13 ; -0.34] | *t*(29) = -5.77, ***p* ≤ .001, *d* = 1.05** $\bar{d}$ = -1.24 [-1.68 ; -0.80] |
| Moderate pain | *t*(29) = -2.00, *p* = .055, *d* = 0.37 $\bar{d}$ = -0.28 [-0.57 ; 0.01] |  | *t*(29) = -4.21, ***p* ≤ .001, *d* = 0.77** $\bar{d}$ = -0.91 [-1.35 ; -0.47] |
| High pain | *t*(29) = -1.29, *p* = .207, *d* = 0.24 $\bar{d}$ = -0.12 [-0.30 ; 0.07] | *t*(29) = -2.50, *p* = .018, *d* = 0.46 $\bar{d}$ = -0.57 [-1.03. ; -0.10] | *t*(29) = -2.23, *p* = .034, *d* = 0.41 $\bar{d}$ = -0.41 [-0.78 ; -0.03] |
| Very high pain | *t*(29) ≤ .01, *p* ≥ .999, *d* = 0.00 $\bar{d}$ ≤ 0.01 [-0.13 ; 0.13] | *t*(29) = -3.21, ***p* = .003, *d* = 0.59** $\bar{d}$ = -0.77 [-1.25 ; -0.28] |  |
| **Average second block** | |  |  |
| No pain |  | *t*(29) = -1.81, *p* = .081, *d* = 0.33 $\bar{d}$ = -0.46 [-0.97 ; 0.06] | *t*(29) = -4.58, ***p* ≤ .001, *d* = 0.84** $\bar{d}$ = -0.93 [-1.35 ; -0.52] |
| Low pain | *t*(29) = -0.52, *p* = .604, *d* = 0.10 $\bar{d}$ = -0.03 [-0.16 ; 0.10] | *t*(29) = -2.54, *p* = .017, *d* = 0.46 $\bar{d}$ = -0.66 [-1.18 ; -0.13] | *t*(29) = -4.83, ***p* ≤ .001, *d* = 0.88** $\bar{d}$ = -0.83 [-1.19 ; -0.48] |
| Moderate pain | *t*(29) = -1.55, *p* = .132, *d* = 0.28 $\bar{d}$ = -0.17 [-0.39 ; 0.05] |  | *t*(29) = -3.70, ***p* ≤ .001, *d* = 0.67** $\bar{d}$ = -0.68 [-1.06 ; -0.31] |
| High pain | *t*(29) = 0.46, *p* = .647, *d* = 0.08 $\bar{d}$ = 0.03 [-0.11 ; 0.18] | *t*(29) = -0.65, *p* = .521, *d* = 0.12 $\bar{d}$ = -0.14 [-0.60 ; 0.31] | *t*(29) = -2.03, *p* = .051, *d* = 0.37 $\bar{d}$ = -0.28 [-0.57 ; 0.002] |
| Very high pain | *t*(29) = 1.48, *p* = .149, *d* = 0.27 $\bar{d}$ = 0.07 [-0.03 ; 0.16] | *t*(29) = -1.21, *p* = .235, *d* = 0.22 $\bar{d}$ = -0.38 [-1.01 ; 0.26] |  |

*Note*. Each cell presents the comparison of the indicated, under- or overpredicting, cue with the correctly predicting cue, per stimulus intensity. Significant *p*-values (Bonferroni corrected alpha *p* ≤ .0125) and moderate and larger effects sizes (Cohen’s *d* ≥ 0.5) are printed bold. * Note that these pairwise comparisons were found to be significant with (zero-inflated) beta regression analyses. $\bar{d}$ indicates the estimated mean difference with the 95% confidence interval.

**tableS5.** Full test results pairwise comparisons affective response ratings in study 1.

| **Pain prediction** | **Non-painful stimulus** | **Moderately painful stimulus** | **Very highly painful stimulus** |
| --- | --- | --- | --- |
| No pain | - | *t(*29) = -7.86, ***p* ≤ .001, *d* = 1.44** | *t*(29) = -11.70, ***p* ≤ .001, *d* = 2.14** |
| Low pain | *t*(29) = -0.29, *p* = .771, *d* = 0.05 | *t*(29) = -6.61, ***p* ≤ .001, *d* = 1.21** | *t*(29) = -11.08, ***p* ≤ .001, *d* = 2.02** |
| Moderate pain | *t*(29) = -1.07, *p* = .294, *d* = 0.20 | - | *t*(29) = -5.97, ***p* ≤ .001, *d* = 1.09** |
| High pain | *t*(29) = -3.33, ***p* = .002, *d* = 0.61** | *t*(29) = -6.09, ***p* ≤ .001, *d* = 1.11** | *t*(29) = -2.72, ***p* = .011, *d* = 0.50** |
| Very high pain | *t*(29) = -4.46, ***p* ≤ .001, *d* = 0.81** | *t*(29) = -7.73, ***p* ≤ .001, *d* = 1.41** | - |

*Note*. Each cell presents the comparison of the indicated, under- or overpredicting, cue with the correctly predicting cue, per stimulus intensity. Significant *p*-values (Bonferroni corrected alpha *p* ≤ .0125) and moderate and larger effects sizes (Cohen’s *d* ≥ 0.5) are printed bold.

**tableS6**. Moderation effects of psychological questionnaire and variability scores on effects of pain predictions on pain intensity ratings in study 1.

| **Moderator** | ***Mean*** | ***SD*** | **Cronbach’s alpha** | **Three-way interaction of pain prediction, stimulus intensity, and questionnaire score** |
| --- | --- | --- | --- | --- |
| Trait anxiety (STAI-T) | 34.70 | 7.73 | 0.89 | *F*(5.16, 144.50) = 0.87, *p* = .503, η_g_^2^ ≤ .01 |
| State anxiety (STAI-S) | 30.44 | 6.88 | 0.63 | *F*(5.18, 144.97) = 0.43, *p* = .833, η_g_^2^ ≤ .01 |
| Pain vigilance and awareness (PVAQ)* | 31.23 | 8.13 | 0.76 | *F*(5.15, 144.31) = 0.36, *p* = .880, η_g_^2^ ≤ .01 |
| Interoceptive Awareness – not distracting (MAIA) | 1.75 | 0.95 | 0.92 | *F*(5.17, 144.71) = 0.38, *p* = .869, η_g_^2^ ≤ .01 |
| Interoceptive Awareness – not worrying (MAIA) | 3.09 | 0.84 | 0.83 | *F*(5.18, 144.91) = 0.74, *p* = .597, η_g_^2^ ≤ .01 |
| Interoceptive Awareness – self regulation (MAIA) | 3.11 | 0.95 | 0.84 | *F*(5.20, 145.61) = 0.56, *p* = .741, η_g_^2^ ≤ .01 |
| Interoceptive Awareness – trust (MAIA) | 4.16 | 0.88 | 0.82 | *F*(5.12, 143.44) = 1.13, *p* = .347, η_g_^2^ ≤ .01 |
| Dispositional optimism (LOT-R) | 15.60 | 4.87 | 0.84 | *F*(5.27, 147.51) = 2.32, *p* = .043, η_g_^2^ = .01 |
| Pain rating variability baseline (*R*^2^) | 0.87 | 0.15 | n.a. | *F*(5.06, 141.61) = 1.04, *p* = .396, η_g_^2^ ≤ .01 |
| Informativeness of cues | 1.60 | 1.00 | n.a. | *F*(5.11, 143.08) = 0.54, *p* = .746, η_g_^2^ ≤ .01 |

*Note*. * item 11 of the PVAQ was erroneously missing, consequently the total score ranged from 0 to 75 (instead of 0 to 80). STAI-T = State-Trait Anxiety Inventory full-form trait scale, STAI-S = State-Trait Anxiety Inventory short-form state scale, PVAQ = Pain Vigilance and Awareness Questionnaire, MAIA = Multidimensional Assessment of Interoceptive Awareness, LOT-R = Revised Life Orientation Test.

# Detailed results study 2

**tableS7.** Frequency of informative predictor per answering option in study 2.

|  | **Not at all** | **Slightly** | **Moderately** | **Very** | **Extremely** |
| --- | --- | --- | --- | --- | --- |
| Did you think the cues were an informative predictor of the intensity of the electrical stimuli? | 5 | 7 | 11 | 6 | 0 |

*Note*. Data from 1 participant is missing

**tableS8.** Mean pain expectation ratings and standard deviation (SD) in study 2.

| **Pain prediction** | ***Mean*** | ***SD*** |
| --- | --- | --- |
| No pain to moderate pain | 2.87 | 1.07 |
| Low pain | 2.72 | 0.97 |
| Low pain to high pain | 4.82 | 0.98 |
| Moderate pain | 4.44 | 0.72 |
| Moderate pain to very high pain | 6.42 | 0.93 |
| Low pain | 2.72 | 0.97 |
| Moderate pain | 4.44 | 0.72 |
| High pain | 6.50 | 0.85 |

*Note*. Pain expectations were rated on an 11-point numerical rating scale, where 0 means no pain at all, 2 means low pain, 4 means moderate pain, 6 means high pain, 8 means very high pain, and 10 means the most intense pain imaginable.

**tableS9.** Mean certainty of pain expectation ratings and standard deviation (SD) in study 2.

| **Pain prediction** | ***Mean*** | ***SD*** |
| --- | --- | --- |
| No pain to moderate pain | 6.18 | 1.05 |
| Low pain | 6.00 | 1.56 |
| Low pain to high pain | 5.01 | 1.55 |
| Moderate pain | 6.05 | 1.08 |
| Moderate pain to very high pain | 5.92 | 1.28 |
| Low pain | 6.00 | 1.56 |
| Moderate pain | 6.05 | 1.08 |
| High pain | 6.55 | 1.14 |

*Note*. Certainty of pain expectations was rated on an 11-point numerical rating scale, where 0 means not certain at all and 10 means very certain.

Data of 2 participants are missing due to a technical error

**tableS10.** Mean pain intensity ratings (primary outcome) and standard deviation (SD) in study 2.

|  | **Average across blocks *(primary)*** | | **Average block 1** | | **Average block 2** | | **Average block 3** | |
| --- | --- | --- | --- | --- | --- | --- | --- | --- |
| **Pain prediction** | *Mean* | *SD* | *Mean* | *SD* | *Mean* | *SD* | *Mean* | *SD* |
| No pain to moderate pain | 3.92 | 1.03 | 3.76 | 1.35 | 4.07 | 1.36 | 3.93 | 1.18 |
| Low pain | 4.09 | 1.10 | 3.98 | 1.40 | 4.28 | 1.30 | 4.01 | 1.23 |
| Low pain to high pain | 4.60 | 0.90 | 4.59 | 1.23 | 4.86 | 1.06 | 4.37 | 1.02 |
| Moderate pain | 4.60 | 0.86 | 4.64 | 1.44 | 4.74 | 0.88 | 4.41 | 0.91 |
| Moderate pain to very high pain | 5.38 | 0.90 | 5.27 | 1.25 | 5.52 | 1.17 | 5.34 | 1.05 |
| High pain | 5.50 | 0.97 | 5.46 | 1.27 | 5.62 | 1.07 | 5.41 | 1.15 |

*Note*. Pain intensity was rated on an 11-point numerical rating scale, where 0 means no pain at all, 2 means low pain, 4 means moderate pain, 6 means high pain, 8 means very high pain, and 10 means the most intense pain imaginable.

**tableS11.** Mean affective response ratings and standard deviation (SD) in study 2.

| **Pain prediction** | ***Mean*** | ***SD*** |
| --- | --- | --- |
| No pain to moderate pain | 1.76 | 0.46 |
| Low pain | 1.58 | 0.51 |
| Low pain to high pain | 2.27 | 0.42 |
| Moderate pain | 2.11 | 0.34 |
| Moderate pain to very high pain | 2.48 | 0.55 |
| High pain | 2.45 | 0.52 |

*Note*. Affective responses to experiencing the predicted pain stimuli were measured on a 5-point Likert scale ranging from 0 = very disappointed, 1 = disappointed, 2 = neutral, 3 = relieved, to 4 = very relieved.

**tableS12.** Mean startle reflex EMG responses in mV and standard deviation (SD) in study 2.

| **Pain prediction** | ***Mean*** | ***SD*** |
| --- | --- | --- |
| No pain to moderate pain | 0.004 | 0.003 |
| Low pain | 0.006 | 0.005 |
| Low pain to high pain | 0.007 | 0.005 |
| Moderate pain | 0.005 | 0.003 |
| Moderate pain to very high pain | 0.006 | 0.004 |
| High pain | 0.006 | 0.005 |

*Note*. Data of 9 participants are missing due to technical difficulties. In addition, about 20% of trials were rejected due to noisy baseline.

**tableS13.** Full test results pairwise comparisons pain expectation ratings in study 2.

| **Pain prediction** | **Underprediction *versus* correct prediction** | **Overprediction *versus* correct prediction** | **Imprecise *versus*  precise prediction** |
| --- | --- | --- | --- |
| Imprecise prediction | *t*(29) = 15.29, ***p* ≤ .001, *d* = 2.79** | *t*(29) = -10.10, ***p* ≤ .001, *d* = 1.84** | - |
| Precise prediction | *t*(29) = 13.37, ***p* ≤ .001, *d* = 2.44** | *t*(29) = -14.27, ***p* ≤ .001, *d* = 2.60** | - |
| Underprediction | - | - | *t*(29) = 1.57, *p* = .128, *d* = 0.29 |
| Correct prediction | - | - | *t*(29) = 3.19, ***p* = .003, *d* = 0.58** |
| Overprediction | - | - | *t*(29) = -0.72, *p* = .476, *d* = 0.13 |

*Note.* Significant *p*-values (Bonferroni corrected alpha *p* ≤ .025 for the comparisons of under-/overprediction with correct prediction) and moderate and larger effects sizes (*d* ≥ 0.5) are printed bold.

**tableS14.** Full test results pairwise comparisons certainty of pain expectation ratings in study 2.

| **Pain prediction** | **Underprediction *versus* correct prediction** | **Overprediction *versus* correct prediction** | **Imprecise *versus*  precise prediction** |
| --- | --- | --- | --- |
| Imprecise prediction | *t*(27) = -4.20, ***p* ≤ .001, *d* = 0.79** | *t*(27) = -3.92, ***p* ≤ .001, *d* = 0.74** | - |
| Precise prediction | *t*(27) = 0.25, *p* = .804, *d* = 0.05 | *t*(27) = -3.01, ***p* = .006, *d* = 0.57** | - |
| Underprediction | - | - | *t*(27) = 0.98, *p* = .336, *d* = 0.19 |
| Correct prediction | - | - | *t*(27) = -3.51, ***p* = .002, *d* = 0.66** |
| Overprediction | - | - | *t*(27) = -3.12, ***p* = .004, *d* = 0.59** |

*Note.* Significant *p*-values (Bonferroni corrected alpha *p* ≤ .025 for the comparisons of under-/overprediction with correct prediction) and moderate and larger effects sizes (*d* ≥ 0.5) are printed bold.

**tableS15.** Full test results pairwise comparisons pain intensity ratings (primary outcome) in study 2.

| **Pain prediction *** | **Underprediction *versus* correct prediction** | **Overprediction *versus* correct prediction** |
| --- | --- | --- |
| Average across blocks *(primary)* | *t*(29) = 7.85, ***p* ≤ .001, *d* = 1.43** $\bar{d}$ = 0.60 [0.44 ; 0.75] | *t*(29) = -9.47, ***p* ≤ .001, *d* = 1.73**  $\bar{d}$ = -0.84 [-1.02 ; -0.65] |
| Average block 1 | *t*(29) = 6.76, ***p* ≤ .001, *d* = 1.23**  $\bar{d}$ = 0.75 [0.52 ; 0.98] | *t*(29) = -6.87, ***p* ≤ .001, *d* = 1.25** $\bar{d}$ = -0.74 [-0.97 ; -0.52] |
| Average block 2 | *t*(29) = 5.45, ***p* ≤ .001, *d* = 1.00** $\bar{d}$ = 0.63 [0.39 ; 0.86] | *t*(29) = -6.98, ***p* ≤ .001, *d* = 1.28** $\bar{d}$ = -0.77 [-1.00 ; -0.55] |
| Average block 3 | *t*(29) = 4.33, ***p* ≤ .001, *d* = 0.79** $\bar{d}$ = 0.42 [0.22 ; 0.61] | *t*(29) = -8.98, ***p* ≤ .001, *d* = 1.64** $\bar{d}$ = -0.99 [-1.21 ; -0.76] |

*Note.* * Comparisons are made with ratings of precise & imprecise predictions pooled together. Significant *p*-values (Bonferroni corrected alpha *p* ≤ .025) and moderate and larger effects sizes (Cohen’s *d* ≥ 0.5) are printed bold. $\bar{d}$ indicates the estimated mean difference with the 95% confidence interval.

**tableS16.** Full test results pairwise comparisons affective response ratings in study 2.

| **Pain prediction *** | **Underprediction *versus* correct prediction** | **Overprediction *versus* correct prediction** |
| --- | --- | --- |
|  | *t*(29) = 6.58, ***p* ≤ .001, *d* = 1.20** | *t*(29) = -3.76, ***p* ≤ .001, *d* = 0.69** |

*Note.* * Comparisons are made with ratings of precise & imprecise predictions pooled together. Significant *p*-values (Bonferroni corrected alpha *p* ≤ .025) and moderate and larger effects sizes (*d* ≥ 0.5) are printed bold.

**tableS17**. Moderation effects of psychological questionnaire and variability scores on effects of pain predictions on pain intensity ratings in study 2.

| **Moderator** | ***Mean*** | ***SD*** | **Cronbach’s alpha** | **Three-way interaction of precision, prediction, and questionnaire score** |
| --- | --- | --- | --- | --- |
| Trait anxiety (STAI-T) | 38.67 | 7.25 | 0.90 | *F*(1.85, 51.86) = 0.55, *p* = .566, η_g_^2^ ≤ .01 |
| State anxiety (STAI-S) | 30.33 | 8.46 | 0.76 | *F*(1.82, 50.98) = 1.11, *p* = .332, η_g_^2^ ≤ .01 |
| Pain vigilance and awareness (PVAQ)* | 34.97 | 9.61 | 0.83 | *F*(1.84, 51.50) = 0.06, *p* = .935, η_g_^2^ ≤ .01 |
| Intolerance of uncertainty (IUS) | 29.90 | 6.93 | 0.84 | *F*(1.83, 51.24) = 0.87, *p* = .417, η_g_^2^ ≤ .01 |
| Dispositional optimism (LOT-R) | 15.53 | 3.54 | 0.79 | *F*(1.85, 51.80) = 0.77, *p* = .461, η_g_^2^ ≤ .01 |
| Pain rating variability (*R*^2^) | 0.83 | 0.10 | n.a. | *F*(1.83, 51.11) = 0.72, *p* = .478, η_g_^2^ ≤ .01 |
| Informativeness of cues | 1.62 | 1.01 | n.a. | *F*(1.83, 49.46) = 0.28, *p* = .734, η_g_^2^ *≤* .01 |

*Note*. * item 11 of the PVAQ was erroneously missing, consequently the total score ranged from 0 to 75 (instead of 0 to 80). STAI-T = State-Trait Anxiety Inventory full-form trait scale, STAI-S = State-Trait Anxiety Inventory short-form state scale, PVAQ = Pain Vigilance and Awareness Questionnaire, IUS = Intolerance of Uncertainty Scale, LOT-R = Revised Life Orientation Test.
